# The abiotic and biotic drivers of rapid diversification in Andean bellflowers (Campanulaceae)

**DOI:** 10.1111/nph.13920

**Published:** 2016-03-14

**Authors:** Laura P. Lagomarsino, Fabien L. Condamine, Alexandre Antonelli, Andreas Mulch, Charles C. Davis

**Affiliations:** ^1^ Department of Organismic and Evolutionary BiologyHarvard University HerbariaHarvard UniversityCambridgeMA02138USA; ^2^ Department of Biological and Environmental SciencesUniversity of GothenburgGöteborgSE 405 30Sweden; ^3^ Gothenburg Botanical GardenGöteborgSE 413 19Sweden; ^4^ Senckenberg Biodiversity and Climate Research Centre (BiK‐F)SenckenbergFrankfurt/Main60325Germany; ^5^ Institute for GeosciencesGoethe University FrankfurtFrankfurt/Main60438Germany

**Keywords:** Andes, biodiversity hotspot, climate change, diversification, Lobelioideae, Neotropics, pollination syndromes, rapid radiation

## Abstract

- The tropical Andes of South America, the world's richest biodiversity hotspot, are home to many rapid radiations. While geological, climatic, and ecological processes collectively explain such radiations, their relative contributions are seldom examined within a single clade.
- We explore the contribution of these factors by applying a series of diversification models that incorporate mountain building, climate change, and trait evolution to the first dated phylogeny of Andean bellflowers (Campanulaceae: Lobelioideae). Our framework is novel for its direct incorporation of geological data on Andean uplift into a macroevolutionary model.
- We show that speciation and extinction are differentially influenced by abiotic factors: speciation rates rose concurrently with Andean elevation, while extinction rates decreased during global cooling. Pollination syndrome and fruit type, both biotic traits known to facilitate mutualisms, played an additional role in driving diversification. These abiotic and biotic factors resulted in one of the fastest radiations reported to date: the centropogonids, whose 550 species arose in the last 5 million yr.
- Our study represents a significant advance in our understanding of plant evolution in Andean cloud forests. It further highlights the power of combining phylogenetic and Earth science models to explore the interplay of geology, climate, and ecology in generating the world's biodiversity.

## Abstract 2

See also the Commentary on this article by Hughes, **210**: 1152–1154.

## Introduction

Species‐rich rapid radiations are a conspicuous ecological and evolutionary phenomenon in the Tree of Life. Clades that have undergone such diversification are often documented in insular environments, including islands (Baldwin & Sanderson, 1998; Givnish *et al*., 2009; Lapoint *et al*., 2014), lakes (Wagner *et al*., 2012), and mountains (McGuire *et al*., 2007; Hoorn *et al*., 2013; Hughes & Atchison, 2015; Merckx *et al*., 2015). Although they represent just one‐eighth of terrestrial land surface, mountains are home to one‐third of all species (Antonelli, 2015) and a large number of species‐rich radiations (Hughes & Atchison, 2015; Schwery *et al*., 2015), including some of the fastest diversification rates reported to date (Madriñán *et al*., 2013). Of particular importance are the tropical Andes, which stretch from Venezuela to northern Argentina along the western coast of South America. These incredibly species‐rich mountains (Barthlott *et al*., 2005; Kreft & Jetz, 2007) are home to *c*. 15% of all flowering plant species, half of which are endemic to the region (Myers *et al*., 2000). The extent of this biodiversity is especially striking considering the recency of mountain uplift: despite debate over the precise timing and rates of uplift (Sempere *et al*., 2006; Ehlers & Poulsen, 2009), an increasing body of evidence suggests that > 60% of the current elevation of the central Andes was attained within the last 10 million yr (Myr) (Gregory‐Wodzicki, 2000; Garzione *et al*., 2006, 2008, 2014). Although the onset of Andean orogeny dates to the Paleocene and Eocene (66–33.9 Myr), including in regions as far north as the modern Eastern Cordillera of Colombia (Parra *et al*., 2009), exceptionally rapid surface uplift occurred during the late Miocene (*c*. 10–6 Myr) and early Pliocene (*c*. 4.5 Myr) (Garzione *et al*., 2008; Hoorn *et al*., 2010; Mulch *et al*., 2010). Such mountain building is thought to promote diversification in a variety of ways, including by increasing physiographic heterogeneity, affecting local and regional climate, facilitating the immigration of preadapted species, and creating opportunities for extensive, parallel geographic speciation and adaptive radiation in island‐ and archipelago‐like venues (Hoorn *et al*., 2013; Givnish *et al*., 2014; Mulch, 2016).

The role of the rise of the Andes in the origination of biodiversity has been implicated in clades as diverse as birds (McGuire *et al*., 2014), butterflies (Elias *et al*., 2009), and angiosperms (Hughes & Eastwood, 2006; Madriñán *et al*., 2013). Within angiosperms, previous work has emphasized that the timing and geotemporal trajectories of diversification have been very different across Andean biomes (Pennington *et al*., 2010). While high‐elevation grassland clades are exceptionally young, fast, and species‐rich (e.g. Hughes & Eastwood, 2006), clades in lower‐elevation dry inter‐Andean valleys are much older, slower, and species‐poor (Särkinen *et al*., 2012). Despite their species richness, however, cloud forest biomes have been largely unexplored with respect to angiosperm diversification, although data suggest that they are intermediate in terms of age and tempo (Särkinen *et al*., 2012).

Identifying the causes of species‐rich rapid radiations, such as those that characterize Andean high‐elevation grasslands and cloud forests, is a major goal in ecology and evolutionary biology. While rapid diversification is undoubtedly the product of both abiotic and biotic factors acting on different spatial and temporal scales (Antonelli & Sanmartín, 2011; Ezard *et al*., 2011; Bouchenak‐Khelladi *et al*., 2015), a tendency to explain a given diversity pattern with a single process has persisted until recently. For montane Andean radiations, this was exemplified by invoking molecular divergence time estimates contemporaneous with the final stages of Andean uplift, during which new habitats formed and provided ecological opportunity for diversification (Hughes & Eastwood, 2006; McGuire *et al*., 2014). However, other factors are probably at play simultaneously. For example, rapid plant diversification in the region has also been attributed to specialized pollination relationships with hummingbirds (Schmidt‐Lebuhn *et al*., 2007; Abrahamczyk *et al*., 2014; Givnish *et al*., 2014), shifts between pollination syndromes (Kay *et al*., 2005), and the acquisition of fleshy fruits in understory taxa (Smith, 2001; Givnish, 2010). Further, these factors are not likely to be independent of one another, e.g. mountain building affects the local climate (Armijo *et al*., 2015), which, in turn, alters biotic interactions (Blois *et al*., 2013). Not surprisingly, diversification studies using molecular phylogenies have become more integrated in their scope as methodology improves, and analyzing multiple factors underlying diversification patterns is becoming tractable (Ezard *et al*., 2011; Wagner *et al*., 2012; Givnish *et al*., 2014; Donoghue & Sanderson, 2015; Hughes *et al*., 2015).

**Figure 1:**
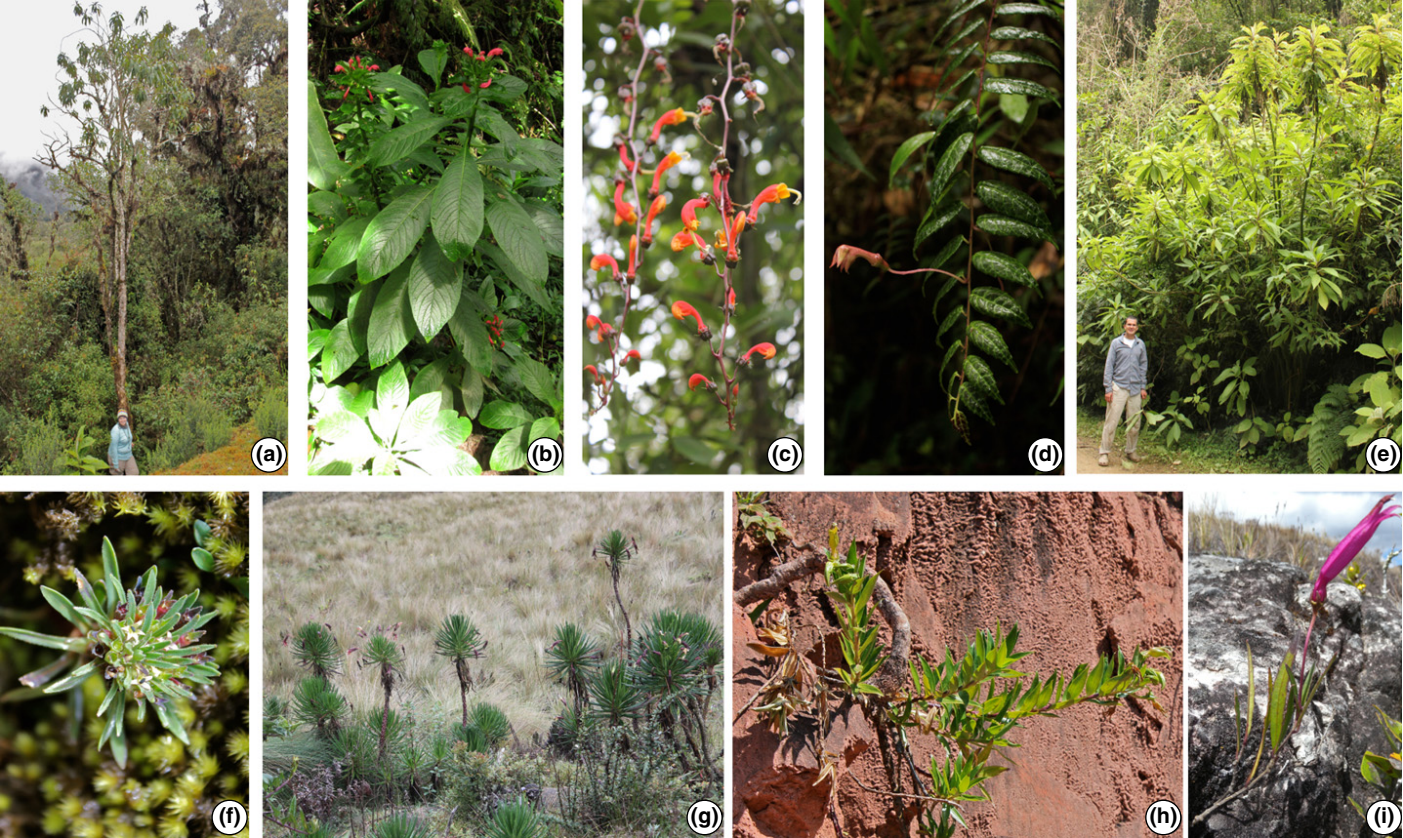
Growth form diversity in Neotropical bellflowers. (a) *Siphocampylus tunarensis* Zahlbr., a tree *c*. 7–10 m. (b) *Centropogon congestus* Gleason, a clonal herbaceous species of wet soil, *c*. 1.5 m. (c) *Centropogon pulcher* Zahlbr., a hanging vine. (d) *Burmeistera* sp., a hemi‐epiphytic herb. (e) *Siphocampylus tunicatus* Zahlbr., a tall shrub, with individual stems *c*. 5 m tall, arising from a single point. (f) *Lysipomia muscoides* Hook f., a minute rosette herb growing among moss in puna habitat. (g) *Siphocampylus jelskii* Zahlbr., a giant rosette shrub with elongated stems, each *c*. 1–2 m tall, apparently clonal, growing in high‐altitude grasslands. (h) *Siphocamplus smilax* Lammers, a xerophyte with a densely woody stem (probably water‐storing) growing in sandstone. (i) *Siphocampylus williamsii* Rubsy, a plant producing a small number of narrow aerial stems arising from a xylopodium, or underground tuber‐like stem. Photographs by: L. Lagomarsino (b–h), D. Santamaría‐Aguilar (a), and A. Fuentes (i).

**Figure 2:**
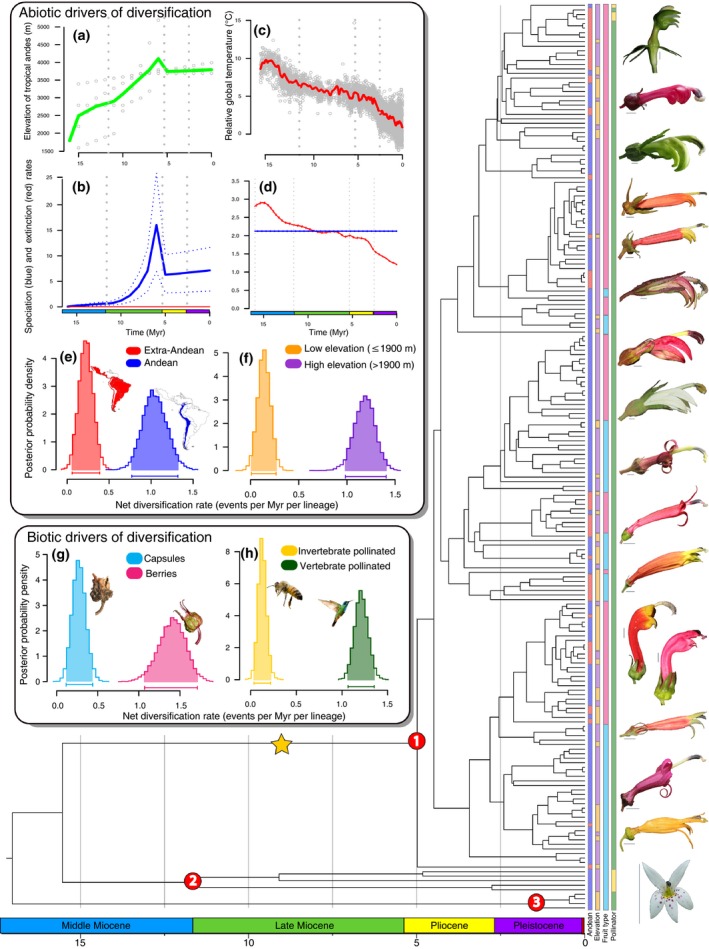
Diversification of Neotropical bellflowers. A time‐calibrated species‐level phylogeny shows the *c*. 5 Myr age of the largely Andean centropogonid clade (*c*. 550 species, node 1), whose origin is associated with a significant increase in diversification rate (yellow star) as detected by Bayesian Analysis of Macroevolutionary Mixture (BAMM). *Lysipomia* (*c*. 50 species) and Chilean *Lobelia* (four species) are indicated (nodes 2 and 3, respectively). Representative floral diversity, shown on the right, illustrates the striking phenotypic diversity in the clade (see Fig. 1 for comparable growth form diversity); scale bars, 0.5 cm. Diversification models examining the abiotic correlates of this rapid diversification include average paleoelevation of the tropical Andes through time (a) and global temperatures through time (c). Gray dots in (a) and (c) represent individual data points utilized to create the curves. Results from these models show inferred speciation (blue) and extinction (red) rates through time under models depending on paleoelevation (b) and paleotemperature (d). Additional diversification analyses using binary state‐speciation and extinction (BiSSE) demonstrate the effect of two abiotic and two biotic traits on net diversification rate: Andean occurrence (e; extra‐Andean (red) vs Andean (blue)), elevation (f; low elevation, ≤ 1900 m (orange) vs high elevation, > 1900 m (purple)), fruit type (g; dry capsules (light blue) vs fleshy berries (pink)), and pollinator type (h; invertebrate (yellow) vs vertebrate (green)). Trait scorings are color‐coded to the right of phylogeny. Outgroups were removed, and taxon names omitted because of space constraints. Photographs by L. Lagomarsino. Geological timescale shown at bottom from Walker *et al*. (2012).

Here, we seek to untangle the ecological and historical factors that have contributed to the generation of Andean megadiversity, especially in mesic mid‐montane forests, using the Neotropical bellflowers as a model system. The Neotropical bellflowers (Campanulaceae: Lobelioideae) are an Andean‐centered (Gentry, 1982) clade of *c*. 600 morphologically and ecologically diverse species found from lowland Amazonia to high‐elevation grasslands above 5000 m, although the vast majority of species are found in cloud forests (Lagomarsino *et al*., 2014). The group was recently resolved into three well‐supported subclades: the Chilean lobelias (*Lobelia* section *Tupa*; four species) of the temperate southern Andes; the páramo and puna endemic *Lysipomia* (50 species); and the centropogonid clade of primarily cloud forest endemics (*Centropogon*,* Burmeistera*, and *Siphocampylus*;* c*. 550 species) (Lagomarsino *et al*., 2014). *Lysipomia* are diminutive herbs, while the other subclades are robust and mostly woody. The centropogonids are particularly diverse in their growth form: most species are vines with woody bases, but herbs, shrubs, and trees are all represented (Fig. 1). Similar phenotypic diversity is also apparent in two traits known to facilitate plant–animal interactions: fruit type and floral morphology. Abiotically dispersed capsules characterize *c*. 40% of the species, while the remaining species produce fleshy, animal‐dispersed berries, which evolved at least seven times from capsular‐fruited ancestors (Lagomarsino *et al*., 2014). Floral morphology is also highly variable. *Lysipomia* produces small, white flowers indicative of invertebrate pollination (Faegri & van der Pijl, 1979), while both the Chilean lobelias and the centropogonids produce robust flowers that are usually vertebrate‐pollinated. Flowers in the centropogonid clade are particularly diverse (Fig. 2) and principally adapted to pollination by hummingbirds and nectar bats (Colwell *et al*., 1974; Stein, 1992; Muchhala, 2006; Muchhala & Potts, 2007).

Owing to their broad Andean distribution and remarkable floral and fruit diversity, the Neotropical bellflowers represent a model group to examine the interaction of abiotic and biotic factors that trigger mountain radiations. To accomplish this goal, we develop and apply numerous statistical models to investigate diversification dynamics in the Neotropical bellflowers. Moreover, our analyses are among the first to incorporate geological data into a model of evolutionary diversification (also see Valente *et al*., 2014 and Mulch, 2016). In this framework, we explicitly investigate the influence of geology, climate, and ecological traits on species diversification using the first well‐sampled, time‐calibrated phylogeny of the group.

## Materials and Methods

### Taxon sampling and molecular dataset

One‐third of Andean bellflower species were sampled, including all four species of *Lobelia* section *Tupa*, five of the *c*. 40 species of *Lysipomia*, and 191 species of the *c*. 550 species of the centropogonid clade (Supporting Information Table S1). Our sampling includes representatives from all taxonomic subdivisions of *Centropogon*,* Burmeistera*, and *Siphocampylus* (Lagomarsino *et al*., 2014). We assembled a concatenated molecular matrix that includes seven plastid regions totaling 11 990 bp. Taxon sampling, molecular methods for newly generated sequences, and alignment protocols followed Lagomarsino *et al*. (2014). Outgroup sampling includes broad representation across Lobelioideae. Nine representatives of Campanuloideae were used as outgroups to root the phylogeny and provide appropriate nodes for fossil calibration.

### Phylogenetics and dating

Phylogenies were inferred using maximum likelihood (ML) and Bayesian inference as implemented in RAxML 8.0 (Stamatakis, 2014) and Beast 2.1.3 (Bouckaert *et al*., 2014). Four calibration points were used to estimate divergence times: the fossil seed of †*Campanula paleopyramidalis* as a minimum age constraint of 16 Myr for the most recent common ancestor of *C. pyramidalis* and *C. carpatica* (Cellinese *et al*., 2009; Crowl *et al*., 2014); a geological maximum age constraint of 29.8 Myr, which corresponds to the age of the Kure atoll, the oldest emerged island of the Hawaiian Ridge (Clague, 1996), for the crown group of the endemic Hawaiian clade, encompassing *c*. 125 spp. in six genera represented in our sampling by *Brighamia*,* Cyanea*,* Clermontia* and *Delissea* (Antonelli, 2009); and two secondary age constraints from Bell *et al*. (2010): 41–67 Myr ago for crown group Campanulaceae, and 28–56 Myr ago for crown group Campanuloideae. The fossil record for Campanulaceae is very poor, and †*C. paleopyramidalis*, described from Miocene deposits in the Nowy Sacz Basin in the West Carpathians of Poland (Łancucka‐Srodoniowa, 1977), is the only fossil appropriate for calibration of divergence time estimates in this family (Antonelli, 2009; Cellinese *et al*., 2009). It is assigned as a close relative of *C. pyramidalis* on the basis of their shared reticulate seed coats, a feature uncommon in the genus (Łancucka‐Srodoniowa, 1977; Cellinese *et al*., 2009).

The optimal RAxML tree was dated using penalized likelihood in treePL (Smith & O'Meara, 2012) with hard minimum and maximum age constraints. Confidence intervals were generated using 1000 RAxML bootstrap trees. We then simultaneously inferred phylogenetic relationships and divergence times using both relaxed uncorrelated lognormal and exponential clock models in Beast. A lognormal prior (mean of 1.5 and SD of 1.0) was assigned to the fossil calibration age of 16 Myr, a uniform prior was assigned to the geological age constraint at 29.8 Myr (maximum hard bound), and normal priors were placed on the two secondary age constraints (mean age of 56.0 Myr and SD of 8.3 Myr for Campanulaceae; mean age of 43.0 Myr and SD of 8.0 Myr for Campanuloideae). The dated tree from treePL was specified as the starting tree in each of eight separate Beast runs, which were each conducted for 10 million generations of Markov chain Monte Carlo (MCMC). Convergence was assessed using effective sample size (ESS) values of the runs in Tracer 1.6 (Rambaut *et al*., 2014), applying a cutoff value of 200. The maximum clade credibility tree, including credibility intervals (CIs) for ages and posterior probabilities (PPs) for node support, was then assembled using TreeAnnotator (Bouckaert *et al*., 2014).

Robustness of age estimates was assessed by removing one or a series of calibration points. The following sets of calibration points were used for this purpose: the Campanulaceae secondary constraint only; the fossil and the Campanulaceae secondary age constraint; and the fossil and both secondary age constraints. Divergence time estimation can be sensitive to branch length variation, which is potentially influenced by growth form (Smith & Donoghue, 2008). As they are generally herbaceous and have longer internal branches than their woody relatives, we suspected this may be the case for *Lysipomia*. We thus removed *Lysipomia* species entirely, and re‐estimated molecular divergence times to determine if these differences had an impact on results. We find no evidence that the results are affected by dating method, calibration strategy, or branch length heterogeneity: the 95% CIs of the re‐estimated ages overlap with the 95% CIs from the Beast analysis that we use for diversification analyses (see also Table S2).

### Trait categorization

Species traits were newly obtained from a variety of sources, including: online databases (GBIF, http://www.gbif.org/ and Tropicos, http://www.tropicos.org/), herbarium specimens, taxonomic literature (Wimmer, 1943, 1953), and field collections. Morphological characters and area of occurrence were coded from type specimens available on the JSTOR Global Plants database (http://plants.jstor.org/) for nearly all 594 species, many of which were not represented in our phylogenetic sampling but were used to account for sampling biases. We scored four binary traits related to the biology and geographic occurrence of all Andean bellflower species for our diversification analyses: fruit type (berry or capsule), pollinator type (vertebrate or invertebrate), elevation (median species occurrence ≤ 1900 m vs > 1900 m), and presence/absence in the Andes. Pollinator type was coded according to classical morphological definitions of pollination syndromes (Faegri & van der Pijl, 1979; Muchhala, 2006). Because they are not sensitive to outliers, we used median elevation across all specimen locality data to determine each species value for elevation. We further used the global median of these values across species as our cutoff for high vs low elevation because this resulted in an equal distribution of the character states (high vs low) across the phylogeny, which is ideal for binary state‐speciation and extinction (BiSSE) analyses (Maddison & FitzJohn, 2015). All trait data are provided in Table S3 and in Fig. 2 and are deposited in Dryad (doi: 10.5061/dryad.7h4j).

### Diversification analyses

We applied a series of birth–death models to quantify the effects of abiotic and biotic correlates of speciation and extinction in Neotropical bellflowers. We assessed the robustness of our results in a variety of ways. First, to accommodate phylogenetic and dating uncertainties (except in the case of the diversity‐dependent analyses, which are computationally intensive), we conducted our analyses across 500 randomly sampled trees from the Beast posterior distribution (outgroups removed). Second, we implemented a sampling fraction to account for nonsampled species diversity. We also applied a sampling fraction, including trait data for species not sampled in our phylogeny, to account for any sampling bias in trait space for BiSSE analyses (Maddison *et al*., 2007). Third, for each of our diversification analyses except for those using Bayesian Analysis of Macroevolutionary Mixture (BAMM 2.2.2, Rabosky, 2014), we selected the best‐fitting model by computing the corrected Akaike information criterion (AICc) based on the log‐likelihood and the number of free parameters for each model. The model supported by the lowest AICc was considered best if it was at least two ∆AIC units better than the model with the second lowest score. We also used likelihood ratio tests (significant at *P *<* *0.05) to estimate support for the best‐fitting model when compared with rival models.

The diversification models we apply assume that extinction rates can be estimated from molecular phylogenetic branching patterns, as originally proposed by Nee *et al*. (1994). It is important to note this is a controversial practice (Rabosky, 2010, 2015), especially without incorporating paleontological data (Quental & Marshall, 2010). Despite this, recent studies have suggested that unbiased estimates of extinction rates can be obtained solely from molecular phylogenies (Morlon *et al*., 2011; Beaulieu & O'Meara, 2015). In this spirit, we explore the individual components of diversification rate in our discussion of results, particularly with respect to paleoenvironmental change.

### Time‐dependent diversification

We used BAMM to estimate speciation and extinction rates through time and to identify shifts in diversification rate. We accounted for incomplete taxon sampling by applying clade‐specific sampling fractions in each of the three principal Andean bellflower subclades: the Chilean lobelias (1.0), *Lysipomia* (0.12), and the centropogonids (0.35). We were unable to provide finer‐scale sampling fractions within the centropogonids because of nonmonophyly of genera and the difficulty of placing species into their constituent clades, particularly within *Siphocampylus* (Lagomarsino *et al*., 2014). While our taxon sampling is fairly low, recent studies exploring the effect of incomplete taxon sampling on the identification of rate shifts suggest that, while results may be incomplete, they should be accurate (Spriggs *et al*., 2015). We ran BAMM with four reversible jump MCMC chains, each for five million generations. ESS values (> 200) were used to assess convergence. The posterior distribution was used to estimate the configuration of the diversification rate shifts, and alternative diversification models were compared using Bayes factors. Results were analyzed and plotted using the R package BAMMtools 2.0.2 (Rabosky *et al*., 2014).

### Paleoenvironment‐dependent diversification

We quantified the effect of the past environment (i.e. climate change and Andean surface uplift) on speciation and extinction rates in Neotropical bellflowers via an approach that builds on time‐dependent diversification models (Nee *et al*., 1994; Morlon *et al*., 2011). The method we utilized allows speciation and extinction rates to correlate not only with time, but also with a quantitative external variable that is time‐dependent (see Condamine *et al*., 2013 for details). These paleoenvironment‐dependent birth–death models are implemented in the R package rpanda (Morlon *et al*., 2016). We incorporated two quantitative external paleoenvironmental variables: the global Cenozoic deep‐sea oxygen isotope record as a proxy for global temperature (Zachos *et al*., 2008), and a generalized model of the paleoelevation history of the tropical Andes. The latter was compiled from several references (Garzione *et al*., 2006, 2008, 2014; Ehlers & Poulsen, 2009; Leier *et al*., 2013) and is available on the Dryad repository (doi: 10.5061/dryad.7h4j).

The R package pspline was used to interpolate a smooth line for each environmental variable. This smooth line was sampled during each birth–death modeling process to give the value of the paleoenvironmental variable at each time point. Speciation and/or extinction rates were then estimated as a function of these values along the dated phylogenies according to the parameters of the following models. For both temperature and Andean elevation, we first applied two standard models, i.e. constant speciation without extinction (Yule model) and constant‐rate birth–death, as null references for model comparison. We then applied four models in which the paleoenvironment dependency was exponential (Table S4): speciation rate varies with the environmental variable and no extinction; speciation rate varies with the environmental variable and extinction rate is constant; speciation rate is constant and extinction rate varies with the environmental variable; and both speciation and extinction rates vary with the environmental variable. We repeated these models with linear dependence on the environmental variable (Table S4).

### Trait‐dependent diversification

We modeled the impact of traits on the diversification of Neotropical bellflowers by concurrently estimating their impact on speciation, extinction, and transition rates using BiSSE (Maddison *et al*., 2007). For each of the four traits (Andean occurrence, elevation, pollination syndrome, and fruit type), we evaluated eight BiSSE diversification models of increasing complexity in which speciation, extinction, and transition rates were allowed to either vary or remain equal between traits (Table S5). Analyses were performed using the R package diversitree 0.7‐6 (FitzJohn, 2012). Once the best‐fitting model was selected, CIs for each parameter were estimated for the tree. We used an exponential prior following FitzJohn (2012), and began the MCMC with the ML estimates. We ran MCMC for 20 000 generations and applied a burn‐in of 2000 steps. We then computed the net diversification rates for each trait. Finally, to determine if these traits were correlated, pairwise trait comparisons were performed across a random sampling of 500 trees from the posterior distribution in both a ML and Bayesian framework in BayesTraits 2.0 (Pagel & Meade, 2006). The significance of binary trait dependence was assessed against a rival model where these traits evolved independently using likelihood ratio tests (ML) and Bayes factors of the harmonic mean of likelihood values.

Binary state‐speciation and extinction models have received recent criticism, including high type I error rates (Maddison & FitzJohn, 2015; Rabosky & Goldberg, 2015), especially for trees with fewer than 300 terminals and for traits present in < 10% of taxa (Davis *et al*., 2013). The distribution of characters states is also important; for example, rapid diversification rates in a region of a phylogeny can be erroneously attributed to a particular character state when the trait is characterized by high transition rates (Rabosky & Goldberg, 2015). However, despite these known issues, SSE models remain a viable framework to test the effect of particular traits on species diversification, particularly when these caveats have been mitigated (Ng & Smith, 2014), as we have attempted to do here. Where possible, we also compare the trait‐dependent results with the inferences made with nontrait‐dependent models (i.e. BAMM and RPANDA) to show that that they are consistent.

### Diversity‐dependent diversification

We applied models that allow speciation and extinction to vary according to the number of lineages in the phylogeny. Using the R package DDD 2.7 (Etienne *et al*., 2012), we built two models: speciation declines with diversity, assuming no extinction, and speciation declines with diversity, allowing for extinction. The initial carrying capacity was set to the current species diversity, and the final carrying capacity was estimated according to the model and corresponding parameters. These models were run on the maximum clade credibility (MCC) tree from our Beast analysis.

## Results

### Molecular phylogeny and divergence time estimation

The final alignment for phylogenetic analysis comprised seven loci (*c*. 12 000 bp) sequenced for 275 species (doi: 10.5061/dryad.7h4j) (Table S1). The concatenated Bayesian phylogenetic analysis using the best‐fitting partitioning scheme yielded a highly resolved and strongly supported tree compatible with the ML tree and previously published results (Lagomarsino *et al*., 2014). Sequence divergence, particularly in the centropogonid clade, was low, as reflected by the shallow branching pattern toward the tips. Despite short internal branches, indicative of a recent radiation, the phylogeny is generally well supported: 66% of nodes are recovered with moderate support (PP > 0.90) and 28% are well supported (PP > 0.95). Our results support the monophyly of the subfamilies Campanuloideae and Lobelioideae and the Neotropical bellflowers (the centropogonids, *Lysipomia*, and Chilean lobelias) with maximum nodal support (PP = 1).

Results of the dating analyses are presented in Fig. 2 (see Figs S1 and S2 for the 95% CI for each node). Similar estimates were obtained using different dating methods and with alternative calibration scenarios and settings (Table S2). This suggests that potential biases such as long internal branch lengths, possibly attributed to growth form variation, do not have a large effect on our age estimates. Based on the molecular divergence time estimates from Beast (Bouckaert *et al*., 2014) and treePL (Smith & O'Meara, 2012), we place the origin of the extant Neotropical bellflowers diversity in the Miocene, *c*. 17.1 Myr ago (95% CI: 14.32–20.32 Myr ago). We further found that the crown groups of their three main subclades – Chilean lobelias, *Lysipomia*, and the centropogonids – originated 1.45 Myr ago (95% CI: 0.42–2.78 Myr ago), 11.87 Myr ago (95% CI: 9.00–15.24 Myr ago), and 5.02 Myr ago (95% CI 3.95–6.13 Myr ago), respectively (Figs 2, S1). All branching events in the most species‐rich clade, the centropogonids, occur in the Plio‐Pleistocene (5.3–0.01 Myr ago).

The age inferred for *Lysipomia* is surprisingly old considering that the genus is currently restricted to páramo and puna vegetations, the youngest of Andean biomes (van der Hammen & Cleef, 1986). This older age is potentially an artifact caused by the substantial DNA substitution rate heterogeneity observed between *Lysipomia* and the rest of the phylogeny, as illustrated in the phylogram in Fig. S3. This elevated substitution rate may be explained by *Lysipomia*'s herbaceous habit or high‐altitude occurrence (Lagomarsino *et al*., 2014). It is likely that this substantial rate heterogeneity is not adequately accommodated by the rate smoothing in Beast, as documented in other groups that exhibit between‐clade rate variation (Beaulieu *et al*., 2015). The age estimates for *Lysipomia* are thus potentially overestimated as a result, which may impact diversification analyses. However, we have shown that this rate heterogeneity does not affect the estimated age of the other two major subclades of Neotropical bellflowers, and as such the impact should be minimal (Table S2).

### Diversification analyses

A time‐dependent diversification analysis in BAMM strongly rejects constant diversification. Instead, it indicates that the Neotropical bellflowers underwent a significant shift in net diversification rate coinciding with the recent origin of the centropogonid subclade (*c*. 5 Myr ago), which includes 550 of the 600 species (Figs 2, S4). This analysis further provides evidence for a 3.7‐fold increase in diversification rate (1.83 vs 0.5 events Myr^–1^ per lineage); net diversification rate is similarly high using an alternative metric (Magallón & Sanderson, 2001) (0.82–1.15 events Myr^–1^ per lineage under high (*ε* = 0.90) and low (*ε* = 0) extinction fractions, respectively). As illustrated in Fig. S4(b), the diversification rate for the centropogonid clade is much higher than its background rate.

We then adapted a recently developed paleoenvironment‐dependent diversification model that explicitly accommodates the effect of changing environment through time (Condamine *et al*., 2013) to examine the effect of Andean paleoelevation (Fig. 2a) and past temperature on diversification rate (Fig. 2d). This novel integration of Andean geological data into an analysis of evolutionary diversification reveals that speciation rates in the Neotropical bellflowers are positively correlated with the elevation history of the Andes, while extinction rates appear to be unlinked to mountain uplift (Fig. 2b; Table S4). Here, we reconstruct a continuous increase in speciation rate beginning at *c*. 12 Myr ago (mid‐Miocene) and peaking at *c*. 5 Myr ago (early Pliocene), consistent with our result from BAMM. This coincides with the attainment of maximum paleoelevation of the Andes (> 4000 m), at which point we infer an unprecedented ~15 speciation events Myr^–1^ per lineage for Neotropical bellflowers. Because of the magnitude of this result, we ran additional analyses implementing a trait‐dependent diversification model using BiSSE (Maddison *et al*., 2007). We investigated two traits that serve as a proxy for the effect of Andean orogeny on diversification in this framework: Andean presence (Andean vs extra‐Andean) and elevational distribution (high elevation (> 1900 m) vs low elevation (≤ 1900 m)). The best‐fitting model for these BiSSE analyses inferred significantly higher speciation rates for Andean species and for species occurring at high elevation (Figs 2e,f, S5; Table S5), respectively. The similar results of these BiSSE analyses are potentially due to the correlated nature of elevational distribution and Andean occurrence that we identified in BayesTraits (Pagel & Meade, 2006) (Table S6); this correlation is not surprising given that the Andes represent the majority of the topographic relief across the Neotropical bellflowers’ range. Together, these results are consistent with an integral role of Andean surface uplift as a trigger of rapid diversification for Neotropical bellflowers.

Using the same paleoenvironmental birth–death model, but instead incorporating relative global temperature proxies that span the last *c*. 20 Myr of the Cenozoic (Zachos *et al*., 2008), we demonstrate a significant correlation between climate change and Andean bellflower diversification (Fig. 2d). In contrast to Andean orogeny, which influenced primarily speciation, the best‐fitting paleoclimatic model suggests that climate change had a significant impact only on extinction. Beginning *c*. 15 Myr ago (mid‐Miocene), extinction rates decreased dramatically towards the present as a function of global temperature (Fig. 2d; Table S4).

Additional BiSSE analyses demonstrate that various clade‐specific biotic traits influence diversification rates in Neotropical bellflowers. BayesTraits analyses showed uncorrelated evolution of these traits with respect to one another, and with both the abiotic factors discussed earlier (Table S6). The best models, as supported by both AICc and likelihood ratio tests (Table S5), infer higher net diversification rates in lineages with two character states: fleshy fruits (vs dry fruits; Figs 2g, S5), and vertebrate pollination syndromes (vs invertebrate pollination syndromes; Figs 2h, S5). These traits acted differently on the two components of diversification: fleshy fruits confer lower extinction rates than capsules, while species pollinated by vertebrates have higher speciation rates than invertebrate‐pollinated species.

Finally, a diversity‐dependent analysis inferred that the Neotropical bellflowers have not reached diversity equilibrium: the estimated carrying capacity (i.e. number of species potentially sustained in the clade) far exceeds the current extant diversity of the clade (Table S7).

## Discussion

The Andes are famous for their high species richness (Myers *et al*., 2000) and the rapid diversification rates that characterize many of their emblematic clades (Hughes & Eastwood, 2006; Madriñán *et al*., 2013; Givnish *et al*., 2014). Although numerous studies have documented this pattern, there have been few attempts to ascertain the processes that underlie this megadiversity, especially in the cloud forests where Neotropical bellflower diversity is concentrated. Our study tackles this issue with an interdisciplinary approach that paves the way for future inquiry.

### The impact of Andean orogeny and climate change on species diversification

Within the morphologically and ecologically diverse Neotropical bellflower clade, a shift to increased diversification rates on the branch subtending the centropogonid subclade resulted in a cloud forest‐centered radiation with *c*. 550 extant species. The diversification rate for the centropogonids, estimated to be between 0.65 and 1.42 events Myr^–1^ per lineage, makes them one of fastest species radiations reported to date (Table 1), with average overall rates of speciation exceeding iconic groups ranging from *Espeletia* (Madriñán *et al*., 2013) and hummingbirds (McGuire *et al*., 2014) in the Andes to the silverswords (Baldwin & Sanderson, 1998) and Drosophilidae of Hawaii (Lapoint *et al*., 2014).

**Table 1:** Net diversification rates in the fastest species radiations in the Tree of Life, including the centropogonids

Clade	Area	Organism	Species richness	Age (95% CI)	*r* (*ε* = 0)	*r* (*ε* = 0.5)	*r* (*ε* = 0.9)
*Dianthus* (carnations) (Valente *et al*., 2010)	Eurasia	Plant	200	1.5 (0.9–2.1)	3.07 (2.12–5.12)	2.88 (2.06–4.80)	1.99 (1.42–3.32)
African cichlids (Salzburger *et al*., 2005)	East African Lakes	Fishes	1800	2.4 (1.22–4.02)	2.83 (1.69–5.76)	2.71 (1.62–5.34)	2.14 (1.28–4.22)
*Lupinus* (Andean clade) (Hughes & Eastwood, 2006)	Andes	Plant	81	1.47 (1.18–1.76)	2.52 (2.10–3.14)	2.33 (1.95–2.90)	1.46 (1.22–1.82)
*Neo‐astragalus* (clade F) (Scherson *et al*., 2008)	Andes	Plant	90	1.89 (1.71–2.07)	2.04 (1.84–2.23)	1.87 (1.70–2.06)	1.19 (1.08–1.31)
*Zosterops* (Moyle *et al*., 2009)	Cosmopolitan	Birds	80	1.84 (1.40–1.89)	2.0 (1.95–2.63)	1.85 (1.80–2.44)	1.16 (1.13–1.52)
*Neo‐astragalus* (clade G) (Scherson *et al*., 2008)	Andes	Plant	15	0.98 (0.79–1.17)	2.06 (1.72–2.55)	1.82 (1.52–2.26)	0.84 (0.70–1.04)
*Gentianella* (von Hagen & Kadereit, 2001)	Andes	Plant	170	1.6–3.0	1.48–2.78	1.39–2.6	0.94–1.77
*Cistus* (Guzmán *et al*., 2009)	Mediterranean	Plant	10	1.04 (0.66–1.29)	1.54 (1.25–2.44)	1.35 (1.09–2.13)	0.57 (0.46–0.89)
*Paussus* (ant‐nest beetles) (Moore & Robertson, 2014)	Madagascar	Insects	86	2.6 (1.7–3.6)	1.45 (1.04–2.21)	1.34 (0.97–2.05)	0.85 (0.61–1.29)
**Centropogonids (this study)**	**Andes**	**Plant**	**556**	**5.02 (3.95–6.13)**	**1.12 (0.91–1.42)**	**1.06 (0.87–1.35)**	**0.79 (0.65–1.00)**
Espeletiinae (Madriñán *et al*., 2013)	Andes	Plant	120	4.04 (2.42–5.92)	1.01 (0.69–1.69)	0.944 (0.64–1.58)	0.62 (0.42–1.04)
AMC clade of Hawaiian *Drosophila* (Lapoint *et al*., 2014)	Hawaii	Insect	91	4.40 (3.45–11.82)	0.94 (0.35–1.2)	0.88 (0.33–1.12)	0.58 (0.22–0.74)
*Inga* (Richardson *et al*., 2001)	Neotropics	Plant	300	5.9 (2.0–13.4)	0.85 (0.37–2.5)	0.8 (0.35–2.36)	0.57 (0.25–1.69)
Ruschioideae (Klak *et al*., 2003)	South Africa	Plant	1563	3.8–8.7	0.7–1.75	0.73–1.68	0.58–1.31
Bee hummingbirds (McGuire *et al*., 2014)	Andes	Birds	36	5	0.58	0.53	0.29
Silversword alliance (Baldwin & Sanderson, 1998)	Hawaii	Plants	28	5.2 (4.4–6.0)	0.51 (0.44–60)	0.46 (0.40–0.54)	0.24 (0.21–0.29)
*Costus* subgenus *Costus* (Kay *et al*., 2005)	Neotropics	Plant	51	1.5–7.1	0.46–2.16	0.42–1.98	0.24–1.16
*Lupinus* ‘super radiation’ (Drummond *et al*., 2012)	Western Americas	Plant	196	5.0–13.2	0.35–0.92	0.33–0.86	0.22–0.59
Hawaiian lobeliads (Givnish *et al*., 2009)	Hawaii	Plants	126	13.6 (10.49–16.71)	0.30 (0.25–0.39)	0.28 (0.23–0.37)	0.19 (0.15–0.24)
Hawaiian Drosophilidae (Lapoint *et al*., 2014)	Hawaii	Insect	1000	25.15 (23.90–27.46)	0.25 (0.23–0.26)	0.24 (0.22–0.25)	0.18 (0.17–0.19)
*Plethodon*,* glutinosus* group (Kozak *et al*., 2006)	North America	Amphibian	30	8–11	0.24–0.34	0.22–0.31	0.12–0.16
Hummingbirds (all) (McGuire *et al*., 2014)	Neotropics	Bird	338	22.4 (20.3–24.7)	0.23 (0.21–0.25)	0.22 (0.20–0.24)	0.16 (0.14–0.17)
*Ctenotus* +* Lerista* (Rabosky *et al*., 2007)	Australia	Reptile	174	c. 20	0.22	0.21	0.14

Rates of diversification as calculated using the statistic of Magallón & Sanderson (2001) under low, medium, and high estimated extinction (*ε*) for the centropogonid clade (bold text) rank ordered with other published rapid radiations. All rates in events Myr^–1^ per lineage. Estimates derived from reported ages and species richness from the relevant literature are cited. When possible, 95% confidence intervals (CIs) for ages are reported, except in cases where sufficient data were unavailable.

In addition, our analyses provide key insights into the abiotic and biotic drivers of rapid diversification in the Andes. Using diversification models that incorporate geological data, we demonstrate that mountain building is correlated with increased diversification rates. Specifically, we demonstrate that the final stages of Andean uplift are strongly associated with the origin and rise of prolific speciation rates in the Neotropical bellflowers (Fig. 2b; Table S4). These rates peaked at a remarkable *c*. 15 events Myr^–1^ per lineage, concurrent with the attainment of the maximum past elevations of the Andes. Although not sustained, this diversification rate exceeds the highest reported in plants, including in the Mediterranean carnations (2.2–7.6 events Myr^–1^ per lineage, Valente *et al*., 2010) and Andean lupines (2.49–5.21 events Myr^–1^ per lineage, Hughes & Eastwood, 2006; Drummond *et al*., 2012). These results are corroborated by the shift to faster speciation rates that was detected by BAMM along the branch that subtends the centropogonid clade, during the period of final Andean uplift. Furthermore, BiSSE analyses bolster our findings that the Andes in general are a major driver of species diversification in this clade: Andean taxa have significantly higher net diversification rates than extra‐Andean taxa (Figs 2e, S5; Table S5). Moreover, species diversification is faster at higher elevations (> 1900 m) (Figs 2f, S5: Table S5). The likely driver of this impressive diversification was not paleoelevation *per se* (as would be expected under a predominantly vicariant model of speciation), but rather ecological opportunity triggered by the availability of wet montane forests. These habitats emerged and greatly expanded across the eastern flanks of the Amazon in the late Miocene and early Pliocene during periods of active mountain building (van der Hammen & Cleef, 1986).

We further present evidence that past climate change has independently influenced diversification rates in the Neotropical bellflowers. Our paleotemperature‐dependency analysis shows that, while speciation rates remained high but constant, extinction rates are positively correlated with past temperature. Consequently, inferred extinction rates were lowest during the coolest past intervals, and have generally been declining since the origin of the clade, a result that is particularly striking in today's world of anthropogenic warming. Although our proxy of climate change is global, it is almost certain that cooling since the mid‐Miocene has affected the Andes, especially at mid‐to‐high elevations, where Neotropical bellflowers are most diverse. We hypothesize that Neotropical bellflowers evolved their tolerance to cool habitats in southern, perhaps temperate, regions of South America, similar to where Chilean lobelias now occur, before major tropical Andean surface uplift. This climatic preadaptation may explain their northward expansion and decreased extinction rates as this clade exploited cooler niches associated with the rising Andes. These results significantly improve our understanding of the abiotic determinants that have triggered Andean diversification, and collectively indicate that mountain building and climate are key contributors to increases in diversification rate in Neotropical bellflowers, although they have acted on different components of net diversification.

### Biotic traits that facilitate mutualisms are associated with elevated rates of net diversification

In addition to orogeny and climate change, our trait‐dependent analyses point to a role of species ecology in influencing species diversification in Neotropical bellflowers (Table S5). Specifically, we identify that mutualistic interactions with seed dispersers and pollinators promote diversification. Here, we demonstrate that bird‐dispersed berries and vertebrate‐pollinated flowers are associated with significantly higher rates of diversification than their counterparts (Figs 2g,h, S4; Table S5). Species with berries have a net diversification rate that is *c*. 3.5 times higher than capsular species, whose seeds are abiotically dispersed (Figs 2g, S4). As in other groups, fleshy fruits in Neotropical bellflowers are generally associated with densely forested understory habitats where abiotic dispersal is not as effective as in open habitats, where capsular taxa dominate (Lagomarsino *et al*., 2014). Owing to the limited movement of understory birds, dispersal of fleshy fruits in these habitats is typically across very short distances (Theim *et al*., 2014). When coupled with rare long‐distance dispersal events, these short dispersal distances can lead to rampant allopatric speciation (Givnish *et al*., 2005, 2009; Givnish, 2010). Further, the repeated evolution of berries in the centropogonid clade may be indicative of multiple parallel radiations across the Andes, each with rapid speciation rates, consistent with a model of cordilleran diversification, as recently proposed in bromeliads (Givnish *et al*., 2014).

Pollination by vertebrates appears to have played an even bigger role in species diversification than seed dispersal. Here, we see an approximately sixfold increase in diversification rate relative to invertebrate pollination (Figs 2h, S5; Table S5). Vertebrate pollinators, namely hummingbirds and bats, are hypothesized to be more effective pollinators than invertebrates, in part because they carry higher pollen loads over longer distances (Fleming *et al*., 2009). The increased speciation rates among vertebrate‐pollinated taxa (Table S5; Fig. S5) are a likely result of floral isolation, which allows prezygotic reproductive isolation to be achieved via an interplay of floral morphology and pollinator behavior (Fulton & Hodges, 1999; Muchhala, 2003; Schiestl & Schlüter, 2009). Supporting this, many Andean bellflower species have sympatric distributions that appear to have been facilitated by either shifts between different classes of vertebrate pollinators (Muchhala, 2006) or character partitioning of floral traits in species that share pollinators (Muchhala & Potts, 2007).

Our analyses of biotic traits indicate that ecological factors, particularly mutualisms with other species, constitute additional triggers of rapid diversification rates in Neotropical bellflowers, as has been demonstrated in extra‐Andean plant groups (Weber & Agrawal, 2014). This effect of species ecology is especially pronounced within the centropogonids, in which more than half of the species are vertebrate‐pollinated and/or produce berries, and in which diversification rates are particularly elevated (Fig. S4). Our results further demonstrate that diversity‐dependent processes did not govern diversification, suggesting that the clade is in its early phases of diversification and is not presently bounded by ecological limits.

### A synergy of abiotic and biotic factors drives diversification in Andean cloud forests

Although the interplay between abiotic and biotic drivers has long been recognized as fundamental for regulating diversity, progress toward understanding their interaction has been slow. Our study is among the first to explore this interaction in a clade distributed predominantly in Andean cloud forests. Using the Neotropical bellflowers as a model system, we first provide important evidence that the age and tempo of diversification of cloud forest clades are intermediate between those in the older inter‐Andean valleys and more recent high‐elevation grasslands (Pennington *et al*., 2010; Särkinen *et al*., 2012). We then demonstrate that Andean surface uplift and climate are both significantly correlated with the rapid radiation of Neotropical bellflowers. In the context of Andean biodiversity, dramatic geological and environmental transformations, often resulting in habitat barriers and steep environmental gradients (e.g. high ridges and low valleys), characterized the matrix in which plant lineages and their mutualists diversified. Our results indicate that key differences in biotic traits, including fruit type and pollination syndrome, confer enhanced diversification capacity among closely related species co‐occurring in this rapidly changing environment. We hypothesize that preadaptation to cooler climates as the tropical Andes rose allowed this clade to achieve its broad Andean distribution, centered in wet, mid‐elevation cloud forests. This, in turn, precipitated mutualistic interactions with groups showing similarly high Andean diversity (e.g. hummingbirds), further enhancing diversification. Within the context of fruit type, limited dispersibility coupled with rare long‐distance dispersal events in berry‐producing lineages resulted in increased diversification rates, probably via allopatric speciation, as shown in the closely related Hawaiian lobeliads (Givnish *et al*., 2009). At the same time, an association with vertebrate pollinators resulted in increased rates of speciation via floral isolation. As also demonstrated in bromeliads (Givnish *et al*., 2014), the repeated evolution of fruit types and pollination syndromes in the complex landscape of the Andes led to multiple parallel radiations in Neotropical bellflowers, which together resulted in a very large, very fast radiation: the centropogonid clade. We argue that while the Andes, and the habitats and habitat heterogeneity created by their orogeny, acted as a montane species pump across many clades, it is an additive interaction of ecological and environmental factors underlies this region's megadiversity.

More generally, rapid plant diversification in the world's montane regions is frequently a product of island‐like ecological opportunity following mountain uplift, further stimulated by evolutionary innovation (Hughes & Eastwood, 2006; Hughes & Atchison, 2015). Our analyses demonstrate that this model applies to Neotropical bellflowers, and is likely relevant to other Andean cloud forest clades. However, why are the Andes more diverse than other montane systems? It is likely that the recency of Andean mountain building partially explains this phenomenon: many clades may still be in the early phases of explosive radiation and thus are not yet greatly impacted by extinction. In addition, the Andes are characterized by deeply dissected topography, including some of the steepest environmental gradients known (Hughes & Atchison, 2015). These characteristics, which almost certainly enhance the island‐like nature of this system and further intensify ecological opportunity, are shared with the Hengduan Mountains (Favre *et al*., 2014), another of the world's biodiversity hotspots (Myers *et al*., 2000; Kreft & Jetz, 2007). We are likely to gain insight into why the Andes are so diverse by comparing their clades’ diversification histories with those in other species‐rich montane regions. Our framework, including the integration of climatic, paleoaltimetry, and trait data, can be used towards this end.

## Author contributions

L.P.L., F.L.C., A.A. and C.C.D. designed the study; A.M. compiled the paleoaltimetry data; L.P.L. assembled all other data; L.P.L. and F.L.C. analyzed data; and L.P.L. and C.C.D. wrote the paper, with significant contributions from F.L.C. and A.A. and revisions from A.M.

## Supporting information

Please note: Wiley Blackwell are not responsible for the content or functionality of any supporting information supplied by the authors. Any queries (other than missing material) should be directed to the *New Phytologist* Central Office.

**Fig. S1** Time‐calibrated phylogeny of Neotropical Campanulaceae, with outgroup representatives of Lobelioideae and Campanuloideae collapsed.
**Fig. S2** Time‐calibrated phylogeny of Neotropical Campanulaceae, with the ingroup collapsed and showing outgroup relationships.
**Fig. S3** Phylogram showing molecular‐proportional branch lengths, illustrating substitution rate heterogeneity within clade.
**Fig. S4** Diversification rate analysis for Neotropical Campanulaceae in BAMM.
**Fig. S5** Posterior distributions of trait‐dependent speciation, extinction, transition, and net diversification rates as estimated by BiSSE.
**Table S1** Taxon sampling including voucher information, country of origin, and GenBank accession numbers for seven plastid loci
**Table S2** Divergence time estimation to assess sensitivity to dating method, calibration selection, and branch length heterogeniety
**Table S3** Trait codings for the four traits used in our BiSSE analyses
**Table S4** Paleoenvironmental‐dependent diversification analyses using paleoaltimetry and Cenozoic climate data
**Table S5** Model comparison for the four BiSSE analyses presented in the main text
**Table S6** Correlated evolution of binary traits using BayesTraits
**Table S7** Model comparison for the two diversity‐dependence analyses presented, with mean parameter estimates for each model
