# Supplementary material for: The abiotic and biotic drivers of rapid diversification in Andean bellflowers (Campanulaceae)
Source: New Phytol. 2016 Mar 14;210(4):1430–42. doi: 10.1111/nph.13920 (PMC4950005; doi:10.1111/nph.13920)
Supplement: Supplementary file 1 — Fig. S1 Time‐calibrated phylogeny of Neotropical Campanulaceae, with outgroup representatives of Lobelioideae and Campanuloideae collapsed. Fig. S2 Time‐calibrated phylogeny of Neotropical Campanulaceae, with the ingroup collapsed and showing outgroup relationships. Fig. S3 Phylogram showing molecular‐proportional branch lengths, illustrating substitution rate heterogeneity within clade. Fig. S4 Diversification rate analysis for Neotropical Campanulaceae in BAMM. Fig. S5 Posterior distributions of trait‐dependent speciation, extinction, transition, and net diversification rates as estimated by BiSSE. Table S1 Taxon sampling including voucher information, country of origin, and GenBank accession numbers for seven plastid loci Table S2 Divergence time estimation to assess sensitivity to dating method, calibration selection, and branch length heterogeniety Table S3 Trait codings for the four traits used in our BiSSE analyses Table S4 Paleoenvironmental‐dependent diversification analyses using paleoaltimetry and Cenozoic climate data Table S5 Model comparison for the four BiSSE analyses presented in the main text Table S6 Correlated evolution of binary traits using BayesTraits Table S7 Model comparison for the two diversity‐dependence analyses presented, with mean parameter estimates for each model [file NPH-210-1430-s001.pdf]

New Phytologist **Supporting Information**

Article title: **The abiotic and biotic drivers of rapid diversification in Andean bellflowers (Campanulaceae)**

Authors: Laura P. Lagomarsino, Fabien L. Condamine, Alexandre Antonelli, Andreas Mulch, Charles C. Davis

Article acceptance date: 26 January 2016

The following Supporting Information is available for this article:

Fig. S1 Time-calibrated phylogeny of Neotropical Campanulaceae, with outgroup representatives of Lobelioideae and Campanuloideae collapsed.

Fig. S2 Time-calibrated phylogeny of Neotropical Campanulaceae, with the ingroup collapsed and showing outgroup relationships.

Fig. S3 Phylogram showing molecular-proportional branch lengths, illustrating substitution rate heterogeneity within clade.

Fig. S4 Diversification rate analysis for Neotropical Campanulaceae in BAMM.

Fig. S5 Posterior distributions of trait-dependent speciation (i), extinction (ii), transition (iii), and net diversification rates (iv) as estimated by BiSSE.

Table S1 Taxon sampling including voucher information, country of origin, and GenBank accession numbers for seven plastid loci.

Table S2 Divergence time estimation to assess sensitivity to dating method, calibration selection, and branch length heterogeniety.

Table S3 Trait codings for the four traits used in our BiSSE analyses.

Table S4 Paleoenvironmental-dependent diversification analyses using paleoaltimetry (a) and Cenozoic climate data (b).

Table S5 Model comparison for the four BiSSE analyses presented in the main text.

Table S6 Correlated evolution of binary traits using BayesTraits.

Table S7 Model comparison for the two diversity-dependence analyses presented, with mean parameter estimates for each model.

Figure S1 (next page). Time-calibrated phylogeny of Neotropical Campanulaceae, with outgroup representatives of Lobelioideae and Campanuloideae collapsed. Phylogeny represents a maximum clade credibility tree resulting from BEAST analysis. Bars at nodes represent the 95% highest posterior density (HPD) of inferred ages. Time series below the chronogram is in Ma. Ingroup relationships shown, with outgroup clades collapsed. Red arrow marks the ingroup, the Neotropical bellflowers. Taxon names are color-coded accorded to current taxonomy: *Burmeistera* (green), *Centropogon* (yellow), *Siphocampylus* (red), *Lysipomia* (purple), and *Lobelia* section *Tupa* (light blue).

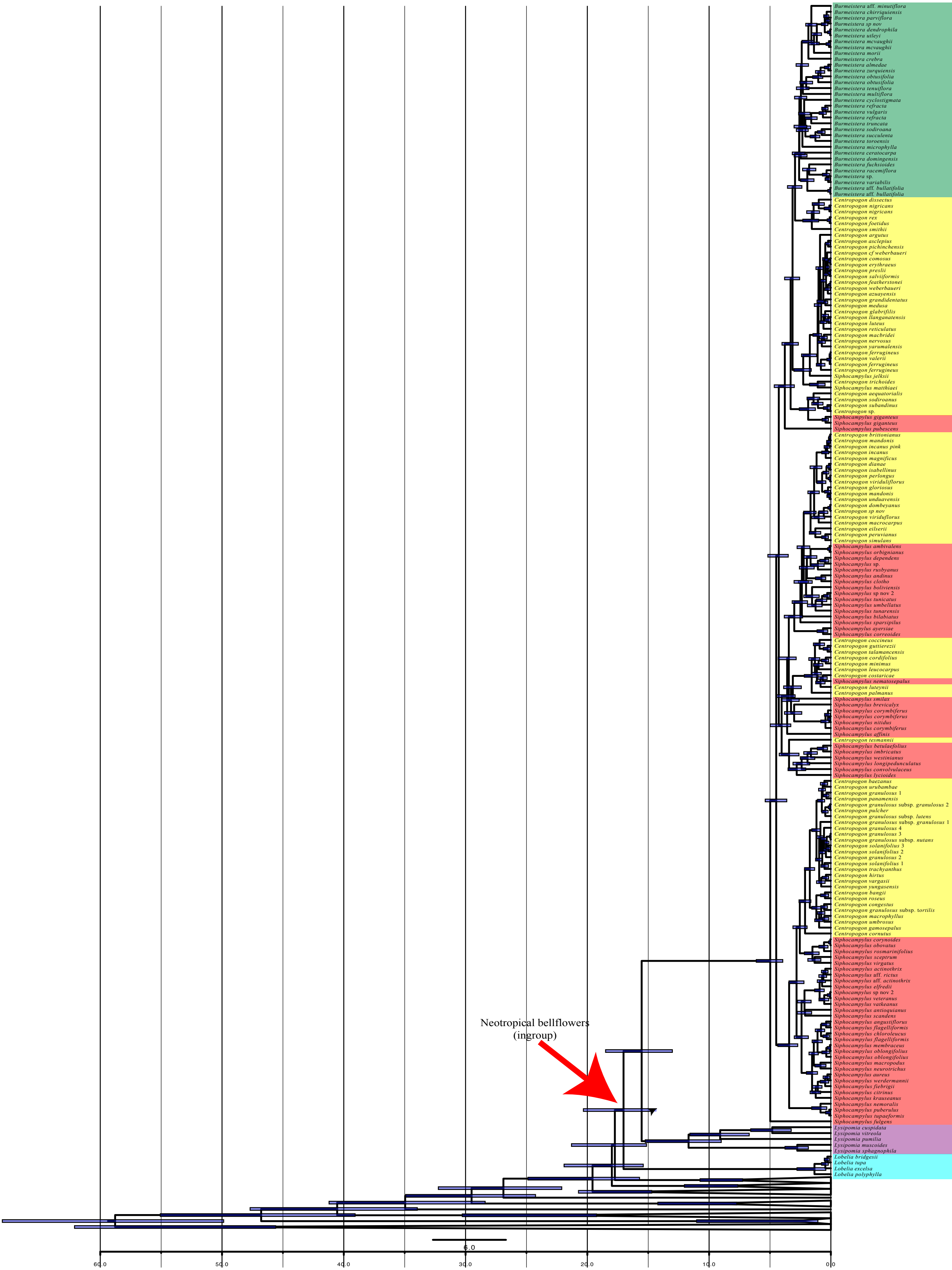

Burmeistera aff. minutiflora
Burmeistera chiriquiensis
Burmeistera parviflora
Burmeistera sp nov
Burmeistera dendrophila
Burmeistera utleyi
Burmeistera mcvaughii
Burmeistera mcvaughii
Burmeistera morii
Burmeistera crebra
Burmeistera almedae
Burmeistera zurquiensis
Burmeistera obtusifolia
Burmeistera obtusifolia
Burmeistera tenuiflora
Burmeistera multiflora
Burmeistera cyclostigmata
Burmeistera refracta
Burmeistera vulgaris
Burmeistera refracta
Burmeistera truncata
Burmeistera sodiroana
Burmeistera succulenta
Burmeistera toroensis
Burmeistera microphylla
Burmeistera ceratocarpa
Burmeistera domingensis
Burmeistera fuchsioides
Burmeistera racemiflora
Burmeistera sp.
Burmeistera variabilis
Burmeistera aff. bullatifolia
Burmeistera aff. bullatifolia
Centropogon dissectus
Centropogon nigricans
Centropogon nigricans
Centropogon rex
Centropogon foetidus
Centropogon smithii
Centropogon argutus
Centropogon asclepius
Centropogon pichinchensis
Centropogon cf weberbaueri
Centropogon comosus
Centropogon erythraeus
Centropogon preslii
Centropogon salviiformis
Centropogon featherstonei
Centropogon weberbaueri
Centropogon azuayensis
Centropogon grandidentatus
Centropogon medusa
Centropogon glabrifilis
Centropogon llanganatensis
Centropogon luteus
Centropogon reticulatus
Centropogon macbridei
Centropogon nervosus
Centropogon yarumalensis
Centropogon ferrugineus
Centropogon valerii
Centropogon ferrugineus
Centropogon ferrugineus
Siphocampylus jelskii
Centropogon trichoides
Siphocampylus matthiaei
Centropogon aequatorialis
Centropogon sodiroanus
Centropogon subandinus
Centropogon sp.
Siphocampylus giganteus
Siphocampylus giganteus
Siphocampylus pubescens
Centropogon brittonianus
Centropogon mandonis
Centropogon incanus pink
Centropogon incanus
Centropogon magnificus
Centropogon dianae
Centropogon isabellinus
Centropogon perlongus
Centropogon viriduliflorus
Centropogon gloriosus
Centropogon mandonis
Centropogon unduavensis
Centropogon dombeyanus
Centropogon sp nov
Centropogon viridiflorus
Centropogon macrocarpus
Centropogon eilserii
Centropogon peruvianus
Centropogon simulans
Siphocampylus ambivalens
Siphocampylus orbignianus
Siphocampylus dependens
Siphocampylus sp.
Siphocampylus rusbyanus
Siphocampylus andinus
Siphocampylus clotho
Siphocampylus boliviensis
Siphocampylus sp nov 2
Siphocampylus tunicatus
Siphocampylus umbellatus
Siphocampylus tunarensis
Siphocampylus bilabiatus
Siphocampylus sparsipilus
Siphocampylus ayersiae
Siphocampylus correoides
Centropogon coccineus
Centropogon guttierezii
Centropogon talamancensis
Centropogon cordifolius
Centropogon minimus
Centropogon leucocarpus
Centropogon costaricae
Siphocampylus nematosepalus
Centropogon luteynii
Centropogon palmanus
Siphocampylus smilax
Siphocampylus brevicalyx
Siphocampylus corymbiferus
Siphocampylus corymbiferus
Siphocampylus nitidus
Siphocampylus corymbiferus
Siphocampylus affinis
Centropogon tesmannii
Siphocampylus betulaefolius
Siphocampylus imbricatus
Siphocampylus westinianus
Siphocampylus longipedunculatus
Siphocampylus convolvulaceus
Siphocampylus lycioides
Centropogon baezanus
Centropogon urubambae
Centropogon granulosus 1
Centropogon panamensis
Centropogon granulosus subsp. granulosus 2
Centropogon pulcher
Centropogon granulosus subsp. lutens
Centropogon granulosus subsp. granulosus 1
Centropogon granulosus 4
Centropogon granulosus 3
Centropogon granulosus subsp. nutans
Centropogon solanifolius 3
Centropogon solanifolius 2
Centropogon granulosus 2
Centropogon solanifolius 1
Centropogon trachyanthus
Centropogon hirtus
Centropogon vargasii
Centropogon yungasensis
Centropogon bangii
Centropogon roseus
Centropogon congestus
Centropogon granulosus subsp. tortilis
Centropogon macrophyllus
Centropogon umbrosus
Centropogon gamosepalus
Centropogon cornutus
Siphocampylus corynoides
Siphocampylus obovatus
Siphocampylus rosmarinifolius
Siphocampylus sceptrum
Siphocampylus virgatus
Siphocampylus actinothrix
Siphocampylus aff. rictus
Siphocampylus aff. actinothrix
Siphocampylus elfredii
Siphocampylus sp nov 2
Siphocampylus veteranus
Siphocampylus vatkeanus
Siphocampylus antioquianus
Siphocampylus scandens
Siphocampylus angustiflorus
Siphocampylus flagelliformis
Siphocampylus chloroleucus
Siphocampylus flagelliformis
Siphocampylus membraceus
Siphocampylus oblongifolius
Siphocampylus oblongifolius
Siphocampylus macropodus
Siphocampylus neurotrichus
Siphocampylus aureus
Siphocampylus werdermannii
Siphocampylus fiebrigii
Siphocampylus citrinus
Siphocampylus krauseanus
Siphocampylus nemoralis
Siphocampylus puberulus
Siphocampylus tupaeformis
Siphocampylus fulgens
Lysipomia cuspidata
Lysipomia vitreola
Lysipomia pumilia
Lysipomia muscoides
Lysipomia sphagnophila
Lobelia bridgesii
Lobelia tupa
Lobelia excelsa
Lobelia polyphylla
Neotropical bellflowers
(ingroup)
6.0
60.0
50.0
40.0
30.0
20.0
10.0
0.0

Figure S2 (next page). Time-calibrated phylogeny of Neotropical Campanulaceae, with the ingroup collapsed and showing outgroup relationships. Phylogeny represents a maximum clade credibility tree resulting from BEAST analysis. Bars at nodes represent the 95% highest posterior density (HPD) of inferred ages. Time series below the chronogram is in Ma. Outgroup relationships, with ingroup collapsed (in red, at top). Red arrows denote major taxonomic groups: Campanulaceae, Campanuloideae, and Lobelioideae. Numbers circled at nodes correspond to the four calibration points used to infer ages: 1) secondary age constraint for crown group Campanulaceae; 2) secondary age constraint for crown group Campanuloideae; 3) the fossil, *Campanula paleopyramidalis* at most recent common ancestor of *C. pyramidalis* Gilib. and *C. carpatica* Jacq.; and 4) the geological age constraint for the oldest island of the Hawaiian Ridge. See methods in main text for additional details.

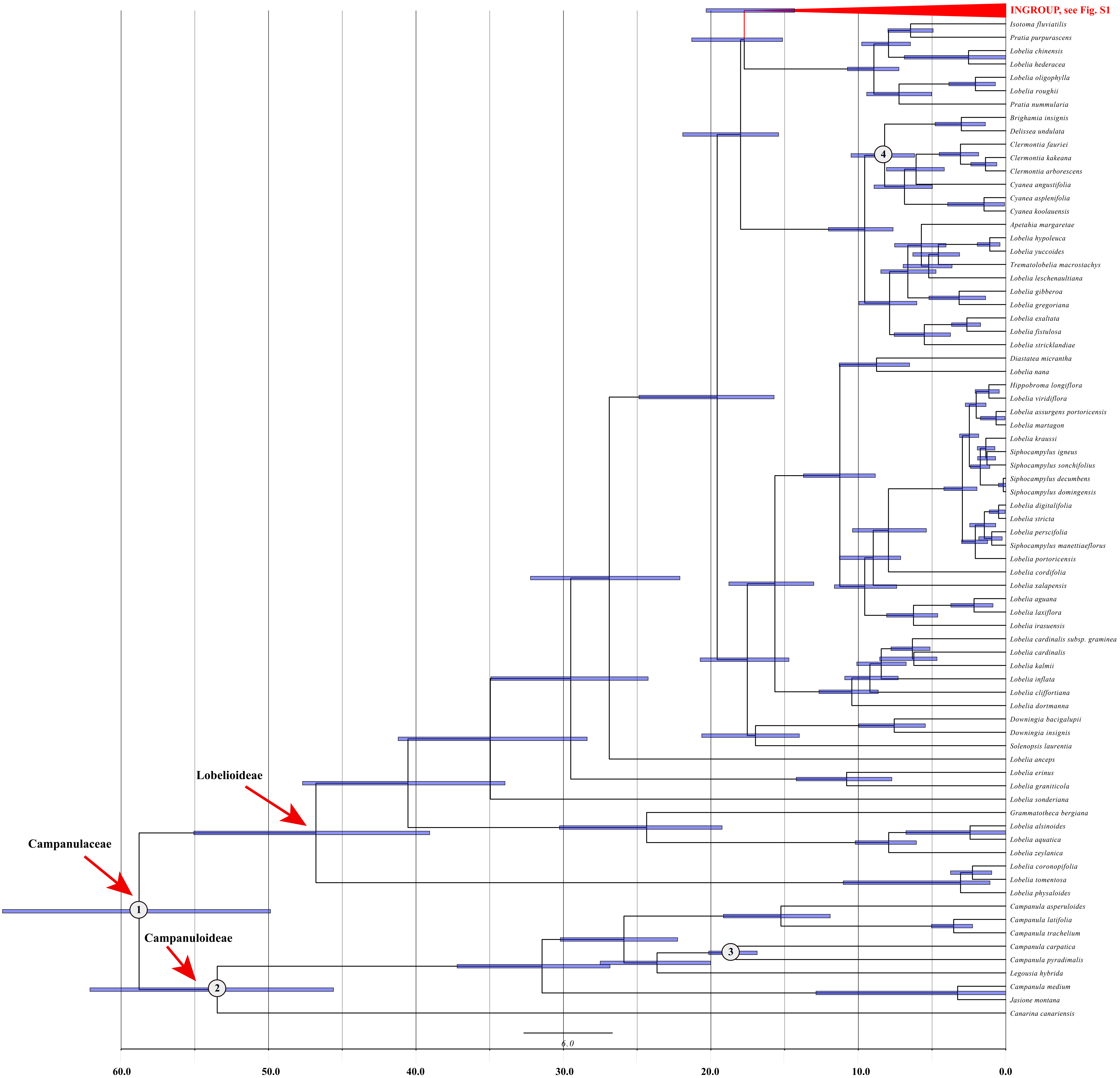

INGROUP, see Fig. S1
Isotoma fluviatilis
Pratia purpurascens
Lobelia chinensis
Lobelia hederacea
Lobelia oligophylla
Lobelia roughii
Pratia nummularia
Brighamia insignis
Delissea undulata
Clermontia fauriei
Clermontia kakeana
Clermontia arborescens
Cyanea angustifolia
Cyanea asplenifolia
Cyanea koolauensis
Apetahia margaretae
Lobelia hypoleuca
Lobelia yuccoides
Trematolobelia macrostachys
Lobelia leschenaultiana
Lobelia gibberoa
Lobelia gregoriana
Lobelia exaltata
Lobelia fistulosa
Lobelia stricklandiae
Diastatea micrantha
Lobelia nana
Hippobroma longiflora
Lobelia viridiflora
Lobelia assurgens portoricensis
Lobelia martagon
Lobelia kraussi
Siphocampylus igneus
Siphocampylus sonchifolius
Siphocampylus decumbens
Siphocampylus domingensis
Lobelia digitalifolia
Lobelia stricta
Lobelia perscifolia
Siphocampylus manettiaeflorus
Lobelia portoricensis
Lobelia cordifolia
Lobelia xalapensis
Lobelia aguana
Lobelia laxiflora
Lobelia irasuensis
Lobelia cardinalis subsp. graminea
Lobelia cardinalis
Lobelia kalmii
Lobelia inflata
Lobelia cliffortiana
Lobelia dortmanna
Downingia bacigalupii
Downingia insignis
Solenopsis laurentia
Lobelia anceps
Lobelia erinus
Lobelia graniticola
Lobelia sonderiana
Grammatotheca bergiana
Lobelia alsinoides
Lobelia aquatica
Lobelia zeylanica
Lobelia coronopifolia
Lobelia tomentosa
Lobelia physaloides
Campanula asperuloides
Campanula latifolia
Campanula trachelium
Campanula carpatica
Campanula pyradimalis
Legousia hybrida
Campanula medium
Jasione montana
Canarina canariensis
Lobelioideae
Campanulaceae
Campanuloideae
1
2
3
4
6.0
60.0
50.0
40.0
30.0
20.0
10.0
0.0

Figure S3 Phylogram showing molecular-proportional branch lengths, illustrating substitution rate heterogeneity within clade. Branches colored in red correspond to the centropogonid clade, in blue to *Lysipomia*, and in green to *Lobelia* section *Tupa*; black branches represent species in the outgroup. Note that the branch lengths within the genus *Lysipomia* are much longer than in other parts of the tree, reflecting an elevated substitution rate within this genus, which may result from its minute habit or the extreme high-elevation habitat to which it is endemic. The scale at bottom represents substitutions/site.

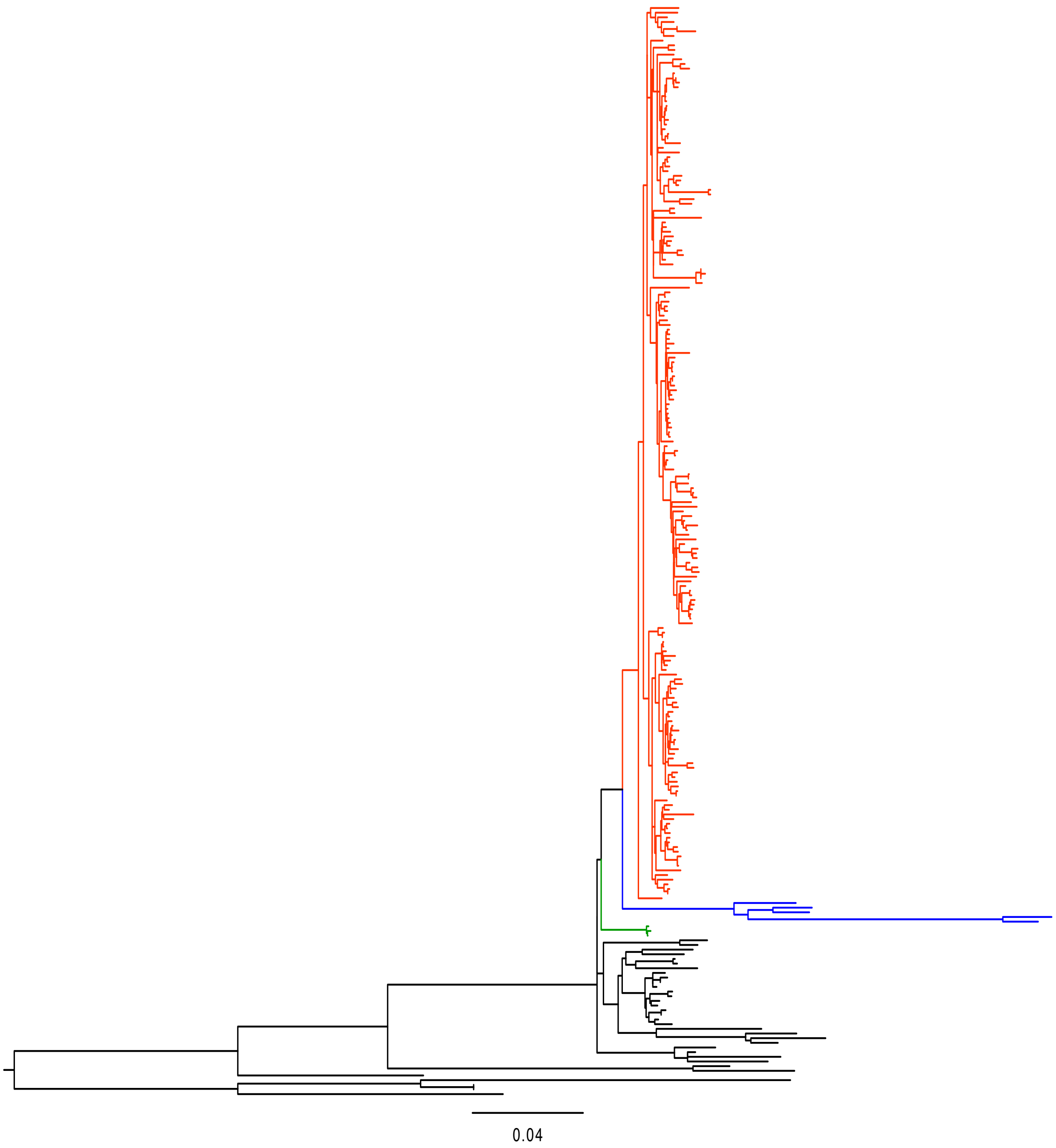

Figure S4. Diversification rate analysis for Neotropical Campanulaceae in BAMM. A. 95% credible set of shift configurations from a BAMM analysis. Colors of branches denote directionality and strength of rate change, cooler and warmer colors designate slower and faster rates, respectively. Significant rate changes are designated by a circle along the branch at which this shift takes place. Note that among the 95% credible set of shift configurations, the majority (~67% of the shift configurations) indicate a significant increase in diversification rate at or near the origin of the centropogonid clade. Bi–iii. Diversification rates (in events/Ma/lineage) through time for the Neotropical bellflower clade (i), Neotropical bellflowers minus the centropogonid clade (ii), and the centropogonid clade alone (iii). Shading in B denotes confidence interval in rate reconstructions.

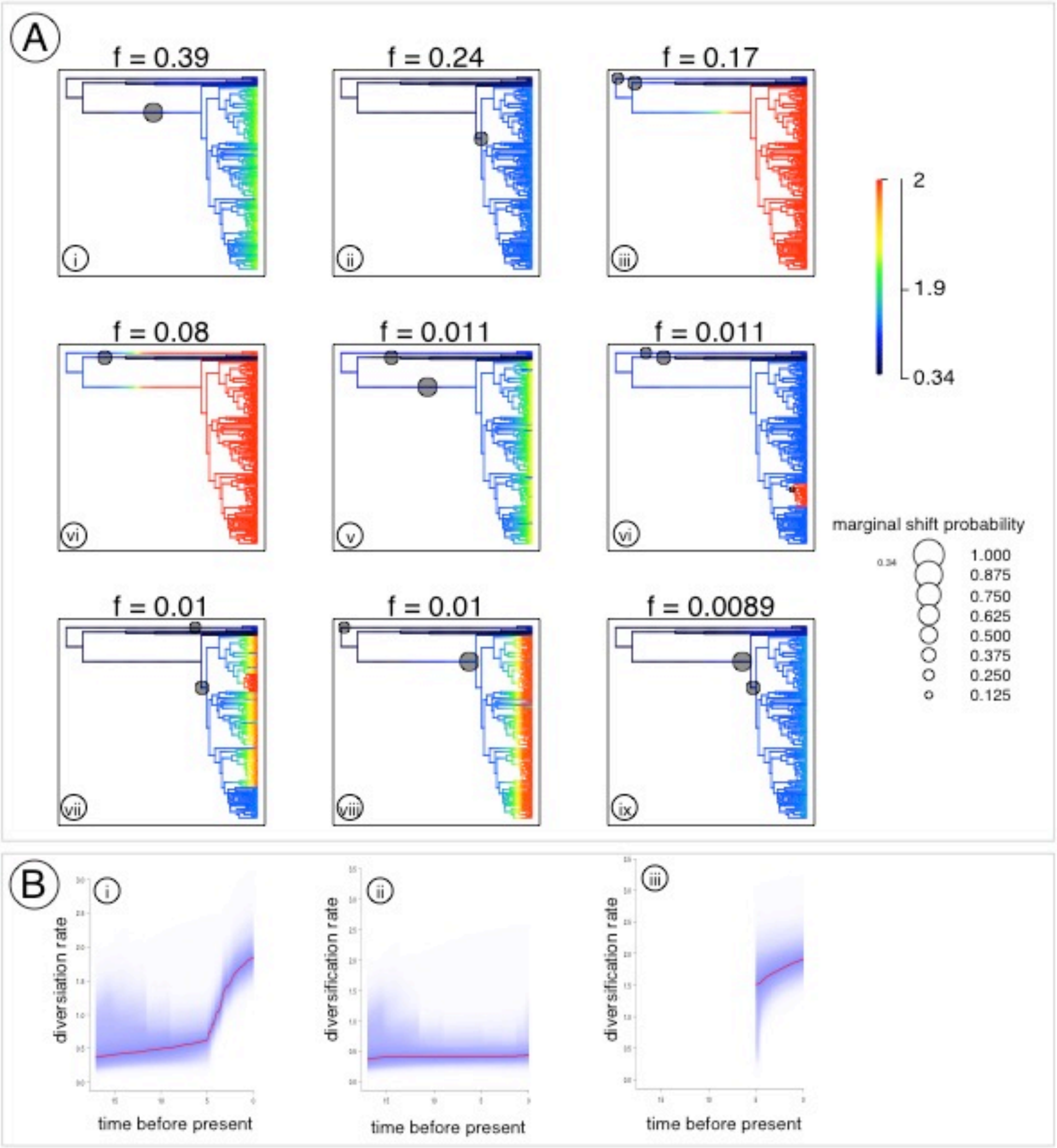

Figure S5. Posterior distributions of trait-dependent speciation (i), extinction (ii), transition (iii), and net diversification rates (iv) as estimated by BiSSE. Histogram colors correspond to Fig. 1 in the main text for each trait, except when the parameter is indistinguishable between the two traits (in which case it is colored gray). A. Andean vs. extra-Andean parameter estimates. B. High vs. low elevation parameter estimates. C. Dry capsule vs. fleshy berry parameter estimates. D. Vertebrate vs. invertebrate pollination parameter estimates.

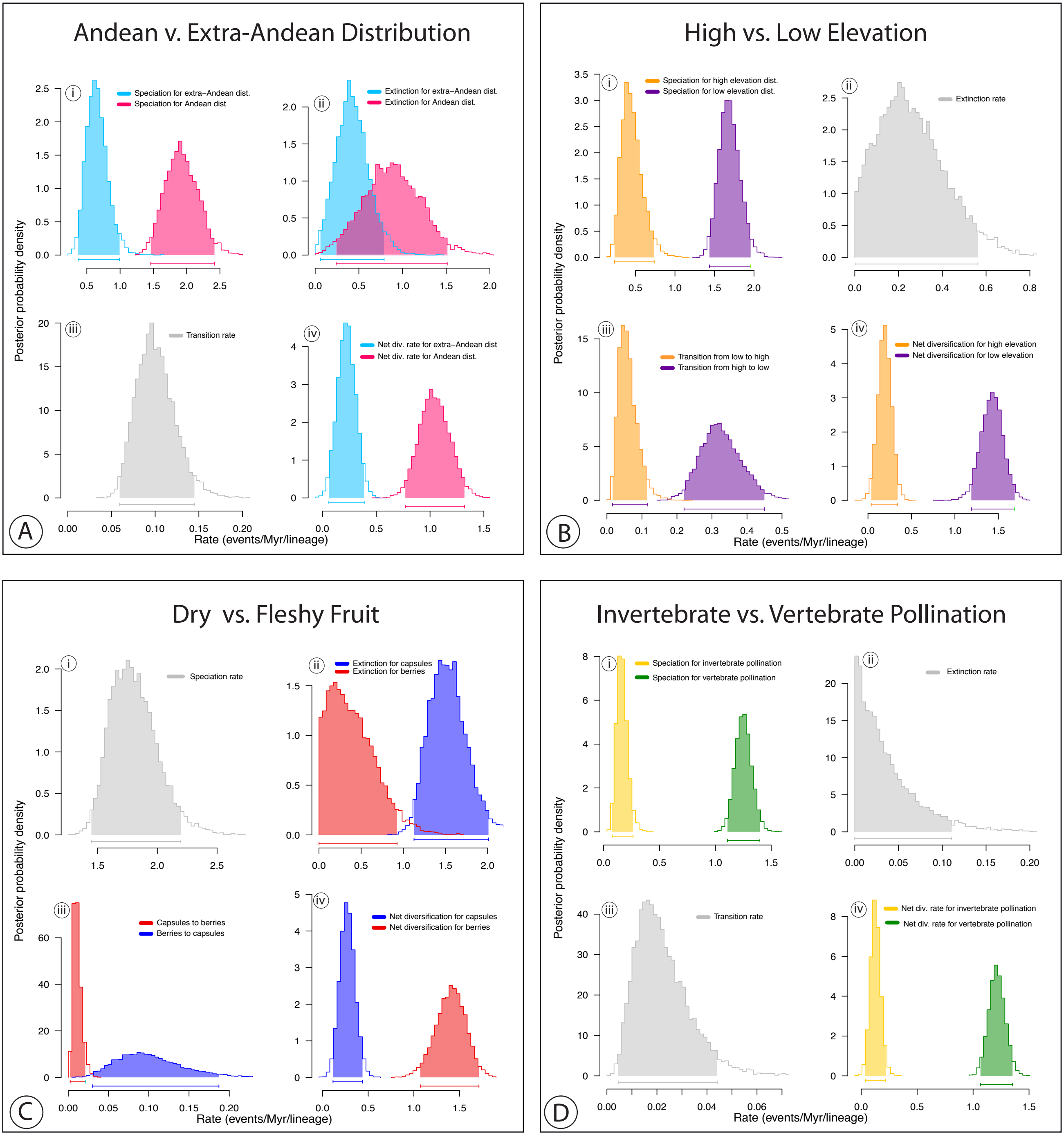
Andean v. Extra-Andean Distribution
Speciation for extra−Andean dist.
Speciation for Andean dist
Extinction for extra−Andean dist.
Extinction for Andean dist.
Transition rate
Net div. rate for extra−Andean dist
Net div. rate for Andean dist.
Posterior probability density
Rate (events/Myr/lineage)
A
High vs. Low Elevation
Speciation for high elevation dist.
Speciation for low elevation dist.
Extinction rate
Transition from low to high
Transition from high to low
Net diversification for high elevation
Net diversification for low elevation
B
Dry vs. Fleshy Fruit
Speciation rate
Extinction for capsules
Extinction for berries
Capsules to berries
Berries to capsules
Net diversification for capsules
Net diversification for berries
C
Invertebrate vs. Vertebrate Pollination
Speciation for invertebrate pollination
Speciation for vertebrate pollination
Extinction rate
Transition rate
Net div. rate for invertebrate pollination
Net div. rate for vertebrate pollination
D

Table S1. Taxon sampling including voucher information, country of origin, and GenBank accession numbers for seven plastid loci.

**Taxon**	**Voucher**	**Origin**	***rpl32-trnL***	***rpl32-ndhF***	***rps16-trnK***	***trnG-trnG-trnS***	***rbcL***	***trnL-trnF***	***ndhF***
INGROUP									
Burmeistera									
Burmeistera almedae Wilbur	L. Lagomarsino 86 (GH)	Costa Rica	KP014195	KP014848	KP014511	KU670709	-	-	-
Burmeistera aff. *bullatifolia* J. Garzón & F. Gonzalez	C. D. Bacon 264A (GB)	Colombia	KP014167	KP014861	KP014501	KU670707	-	-	-
Burmeistera aff. *bullatifoli*a J. Garzón & F. Gonzalez	C. D. Bacon 287 (GB)	Colombia	KP014168	KP014862	KP014502	KP014770	-	-	-
Burmeistera ceratocarpa Zahlb.	M. L. Bristol 993 (GH)	Colombia	KP014172	KP014852	KP014343	-	-	-	-
Burmeistera chiriquiensis Wilbur	D. Santamaría 8996 (INB)	Costa Rica	KP014192	KP014858	KP014521	KP014754	-	-	-
Burmeistera crebra McVaugh	L. Lagomarsino 70 (GH)	Costa Rica	KP014234	KP014870	KP014507	KP014758	-	-	-
Burmeistera cyclostigmata J. D. Smith	L. Lagomarsino 51 (GH); Andersson & Nilsson 2451	Costa Rica	KP014181	KP014876	KP014514	KP014707	DQ356147	DQ356213	DQ356097
Burmeistera dendrophila E. Wimm	L. Lagomarsino 255 (GH)	Panama	KP014163	KP014855	KP014524	KP014750	-	-	-
Burmeistera domingensis Jeppesen	N. Muchhala 102; Harling and Andersson 23154 (GB)	Ecuador	KP014189	KP014860	KP014532	KP014699	EF174633	DQ356210	-
Burmeistera fuchsioides J. Garzón & F. Gonzalez	C. D. Bacon 258 (GB)	Colombia	KP014186	KP014865	KP014503	KU670692	-	-	-
Burmeistera mcvaughii Wilbur	L. Lagomarsino 257 (GH)	Panama	KP014166	KP014853	KP014526	KP014768	-	-	-
Burmeistera mcvaughii Wilbur	L. Lagomarsino 98 (GH)	Panama	KP014233	KP014854	KP014527	KP014769	-	-	-

Burmeistera microphylla J. D. Smith	L. Lagomarsino 34 (GH)	Costa Rica	KP014187	KP014850	KP014350	KP014720	-	-	-
Burmeistera aff. *minutiflora* J. Garzón & F. Gonzalez	C. D. Bacon 262 (GB)	Colombia	KP014235	KP014869	KP014508	KP014712	-	-	-
Burmeistera morii Wilbur	L. Lagomarsino 273 (GH)	Panama	KP014165	KP014864	KP014525	KP014771	-	-	-
Burmeistera multiflora Zahlb.	N. Muchhala 114	Ecuador	KP014160	KP014879	KP014530	KP014583	-	-	-
Burmeistera obtusifolia E. Wimm	L. Lagomarsino 61 (GH)	Costa Rica	KP014197	KP014969	KP014510	KU670700	-	-	-
Burmeistera obtusifolia E. Wimm	L. Lagomarsino 163 (GH)	Panama	KP014196	KP014851	KP014513	KP014701	-	-	-
Burmeistera parviflora E. Wimm	L. Lagomarsino 53 (GH)	Costa Rica	KP014236	KP014859	KP014498	KP014752	-	-	-
Burmeistera racemiflora Lammers	M. Madison 7145 (AAU)	Ecuador	KP014170	KP014845	KP014504	KP014760	-	-	-
Burmeistera refracta E. Wimm.	L. Andersson 2464 (GB)	Ecuador	KP014175	KP014871	KP014493	KP014713	-	-	-
Burmeistera refracta E. Wimm.	N. Muchhala 110	Ecuador	KP014176	KP014880	KP014494	KP014711	-	-	-
Burmeistera sodiroana Zahlb.	N. Muchhala 115	Ecuador	KP014239	KP014867	KP014528	KU67071	-	-	-
Burmeistera succulenta H. Karst. & Triana	C. D. Bacon 297 (GB)	Colombia	KP014240	KP014866	KP014529	KU670697	-	-	-
Burmeistera tenuiflora J.D. Smith	L. Lagomarsino 48 (GH)	Costa Rica	KP014194	KP014923	-	KP014757	-	-	-
Burmeistera toroensis Wilbur	L. Lagomarsino 92 (GH)	Panama	KP014241	KP014868	KP014497	KP014709	-	-	-
Burmeistera truncata Zahlbr.	J. Luteyn 14362	Ecuador	-	-	KU670737	-	-	-	-
Burmeistera utleyi Wilbur	L. Lagomarsino 253 (GH)	Panama	KP014164	KP014856	KP014523	KP014751	-	-	-
Burmeistera variabilis (Gleason) E. Wimm.	C. D. Bacon 298 (GB)	Colombia	KP014169	KP014846	KP014505	KP014759	KU670751	-	-
Burmeistera vulgaris E. Wimm.	D. Santamaría S-980 (GB)	Costa Rica	KP014174	KP014872	KP014495	KP014714	-	-	-
Burmeistera zurquiensis Wilbur	L. Lagomarsino 26 (GH)	Costa Rica	KP014193	KP014849	KP014512	KP014702	KU670805	-	-
Burmeistera sp.	C. D. Bacon 283 (GB)	Colombia	KP014171	KP014844	KP014506	KP014638	-	-	-

Burmeistera sp. nov.	D. Santamaría 6640 (INB)	Costa Rica	KP014237	KP014857	KP014522	KP014753	-	-	-
Centropogon										
Centropogon aequatorialis E. Wimm.	N. Muchhala 131	Ecuador	KP014206	KP014957	KP014531	KP014767	-	-	-
Centropogon argutus E. Wimm.	L. Lagomarsino 329	Peru	KU670740	KU670689	KU670726	KU670711	KU670756	-	-
Centropogon asclepiadeus (Willd.) E. Wimm.	R. E. Weaver, Jr. 1556 (GH)	Colombia	KP014228	KP014927	KP014560	KU670712	-	-	-
Centropogon azuayensis Jeppesen	A. Antonelli 576 (GB)	Ecuador	KP014231	KP014931	KP014558	KU670719	-	-	-
Centropogon baezanus Jeppesen	N. Muchhala 162	Ecuador	KP014141	KP014836	KP014408	KP014624	-	-	-
Centropogon bangii Zahlb.	S. Beck 20763 (OSH)	Bolivia	KP014122	KP014826	KP014409	KP014630	-	-	-
Centropogon brittonianus Zahlb.	L. Lagomarsino 196 (GH)	Bolivia	KP014283	KP015004	KP014477	KP014686	KU670757	-	-
Centropogon coccineus (Hook.) Regel ex B.D. Jacks.	D. Santamaría 8218 (INB)	Costa Rica	KP014304	KP014955	KP014465	KP014763	KU670771	-	-
Centropogon comosus Gleason	P. Nuñéz 8162 (AAU)	Ecuador	KP014229	KP014937	KP014546	KP014739	-	-	-
Centropogon congestus Gleason	L. Lagomarsino 40 (GH)	Costa Rica	KP014127	KP014822	KP014406	KP014595	-	-	-
Centropogon cordifolius Benth.	R. Kriebel 5559 (NY)	Guatemala	KP014253	KP014945	KP014457	KP014773	-	-	-
Centropogon cornutus (L.) Druce	L. Lagomarsino 93 (GH); Jacobs et al. 4955	Panama	KP014121	KP014810	KP014345	KP014593	DQ356158	DQ356226	DQ356106
Centropogon costaricae (Vatke) E. Wimm.	L. Lagomarsino 24A (GH)	Costa Rica	KP014306	KP014949	KP014470	KP014742	KU670774	-	-
Centropogon dianae Lammers	L. Lagomarsino 415 (GH)	Peru	KP014267	KP014988	KP014444	-	KU670769	-	-
Centropogon dissectus E. Wimm.	J. Luteyn 5678 (GB)	Ecuador	KP014212	KP014992	KP014561	KP014722	EF141026	DQ356215	-
Centropogon dombeyanus E. Wimm	L. Lagomarsino 332 (GH)	Peru	-	KP014986	KP014488	KP014663	-	-	-
Centropogon eilersii Lammers & M.O. Dillon	L. Lagomarsino 411 (GH)	Peru	KU670739	KU670685	KU670729	KU670715	-	-	-

Centropogon erythraeus Drake	L. Andersson 2529 (GB)	Ecuador	KP014227	KP014936	KP014559	KP014740	-	-	-
Centropogon featherstonei Gleason	L. Lagomarsino 318 (GH)	Peru	KP014219	KP014933	KP014548	KP014737	KU670758	-	-
Centropogon ferrugineus (L.f) Gleason	D. Santamaría S-902 (GB)	Costa Rica	KP014216	KP014920	KP014543	KP014729	-	-	-
Centropogon ferrugineus (L.f) Gleason	L. Lagomarsino 22 (GH)	Costa Rica	KP014217	KP014925	KP014544	KP014730	KU670759	-	-
Centropogon ferrugineus (L.f) Gleason	L. Lagomarsino 103 (GH)	Panama	KP014203	KP014919	KP014538	KP014696	-	-	-
Centropogon foetidus (Kunth) E. Wimm.	C. D. Bacon 288A (GB)	Colombia	KP014200	KP014971	KP014563	KP014774	-	-	-
Centropogon gamosepalus Zahlb.	N. Muchhala 429; Harling and Andersson 24342	Ecuador	KP014130	KP014823	KP014404	KP014627	DQ356157	DQ356225	DQ356105
Centropogon glabrifilis (E. Wimm.) Jeppsen	L. Andersson 2423 (GB)	Ecuador	KP014222	KP014916	KP014542	KP014728	KU670760	-	-
Centropogon gloriosus (Britton) Zahlb.	L. Lagomarsino 199 (GH)	Bolivia	KP014294	KP014979	KP014451	KP014670	KU670761	-	-
Centropogon grandidentatus (Schltdl.) Zahlbr.	UCBG 81.0952	Cultivated	KU670747	KU670688	KU670721	KU670702	-	-	-
Centropogon granulosus subsp. *granulosus* Stein (ined.) 1	Andersson 2477	Ecuador	KP014136	KU670682	KP014407	KU670701	DQ356152	DQ356220	DQ356101
Centropogon granulosus subsp. *granulosus* Stein (ined.) 2	L. Lagomarsino 330 (GH)	Peru	KP014142	KP014841	KP014413	KP014614	-	-	-
Centropogon granulosus subsp. *lugens* Stein (ined)	C. D. Bacon 303 (GB)	Colombia	KP014123	KP014840	KP014416	KP014618	-	-	-
Centropogon granulosus subsp. *nutans* Stein (ined.)	D. Santamaría S-962 (GB)	Costa Rica	KP014145	KP014830	KP014394	KP014623	-	-	-
Centropogon granulosus subsp. *tortilis* Stein (ined.)	C. Persson 829 (GB)	Panama	KP014126	KP014821	KP014411	KP014605	-	-	-
Centropogon granulosus C. Presl. 1	C. D. Bacon 306 (GB)	Colombia	KU670749	-	KU670725	KU670703	-	-	-
Centropogon granulosus C. Presl. 2	L. Lagomarsino 56 (GH)	Costa Rica	KP014147	KP014833	KP014395	KP014625	-	-	-
Centropogon granulosus C. Presl. 3	L. Lagomarsino 81 (GH)	Costa Rica	KP014144	KP014829	KP014393	KP014622	-	-	-

Centropogon granulosus C. Presl. 4	L. Lagomarsino 301 (GH)	Panama	KP014140	-	KP014396	KP014616	-	-	-
Centropogon gutierrezii (Planch & Oerst.) E. Wimm.	L. Lagomarsino 19 (GH)	Costa Rica	KP014301	KP014950	KP014466	KP014745	-	-	-
Centropogon hirtus (Cav.) C. Presl.	L. Lagomarsino 392	Peru	KU670743	KU670680	KU670727	KU670708	KU670762	-	-
Centropogon incanus (Britton) Zahlb.	S. Acha 207 (MO)	Bolivia	KP014279	KP014998	KP014480	KP014659	-	-	-
Centropogon incanus (Britton) Zahlbr..	L. Lagomarsino 223 (GH)	Bolivia	KP014285	KP014997	KP014476	KP014662	-	-	-
Centropogon isabellinus E. Wimm.	L. Lagomarsino 406 (GH)	Peru	KP014280	KP014983	KP014445	KP014679	-	-	-
Centropogon leucocarpus McVaugh	L. Lagomarsino 280 (GH)	Panama	KP014162	KP014951	KP014462	KP014743	KU670763	-	-
Centropogon llanganatensis Jeppsen	N. Muchhala 139	Ecuador	KP014226	KP014926	KP014556	KP014727	-	-	-
Centropogon luteus E. Wimm.	L. Andersson 2411 (GB)	Ecuador	KP014214	KP014922	KP014555	KP014731	DQ356151	DQ356219	DQ356100
Centropogon luteynii Wilbur	L. Lagomarsino 259 (GH)	Panama	KP014161	KP014911	KP014432	KP014747	KU670764	-	-
Centropogon macbridei Gleason	L. Lagomarsino 337 (GH)	Peru	KP014208	KP014917	KP014540	KP014732	-	-	-
Centropogon macrocarpus Zahlb.	L. Valenzuela 858 (OSH)	Peru	KP014289	-	KP014460	-	-	-	-
Centropogon macrophyllus (G. Don.) E. Wimm	L. Lagomarsino 345 (GH)	Peru	KP014120	KP014824	KP014405	KP014629	-	-	-
Centropogon magnificus Zahlb.	A. Fuentes 10044 (LPB)	Bolivia	KP014288	KP014995	KP014475	KP014658	-	-	-
Centropogon mandonis Zahlb.	A. Timmermann 1 (AAU)	Bolivia	KP014282	KP014999	KP014478	KP014687	-	-	-
Centropogon mandonis Zahlb.	L. Lagomarsino 243 (GH)	Bolivia	KP014295	KP014977	KP014452	KP014666	-	-	-
Centropogon medusa E. Wimm.	N. Muchhala 174	Ecuador	KP014223	KP014930	KP014549	KP014692	KU670765	-	-
Centropogon minimus McVaugh	C. D. Bacon 264B (GB)	Colombia	KP014254	KP014947	KP014458	KP014698	-	-	-
Centropogon nervosus Wimm	L. Lagomarsino 407 (GH)	Peru	KP014202	KP014915	KP014539	KP014724	-	-	-
Centropogon nigricans Zahlb.	J. Ramos 7097 (OSH)	Ecuador	KP014209	-	KP014565	-	-	-	-

Centropogon nigricans Zahlb.	N. Muchhala 118	Ecuador	KP014210	KP015007	KP014566	KP014723	-	-	-
Centropogon palmanus (J. D. Smith) E. Wimm.	L. Lagomarsino 193a (GH)	Costa Rica	KP014303	KP014843	KP014746	KP014746	-	-	-
Centropogon panamensis Wilbur	L. Lagomarsino 308 (GH)	Panama	KP014131	KP014828	KP014403	KP014613	-	-	-
Centropogon perlongus Gleason	L. Lagomarsino 409 (GH)	Peru	KP014265	KP014989	KP014447	KP014681	-	-	-
Centropogon peruvianus (E. Wimm.) McVaugh	L. Lagomarsino 386 (GH)	Peru	KU670744	KU670683	KU670723	KU670695	KU670766	-	-
Centropogon pichinchensis Zahlb.	J. Luteyn 14284 (OSH)	Ecuador	KP014220	-	KP014550	-	-	-	-
Centropogon preslii E. Wimm.	L. Andersson 2503 (GB)	Ecuador	KP014225	KP014929	KP014553	KP014734	-	-	-
Centropogon pulcher Zahlb.	L. Lagomarsino 344 (GH)	Peru	KP014143	KP014842	KP014415	KP014615	KU670767	-	-
Centropogon reticulatus Drake	M. Merello 1099 (MO)	Peru	KP014185	KP014921	KP014557	KP014718	-	-	-
Centropogon rex E. Wimm.	C. D. Bacon 288 (GB)	Colombia	KP014201	KP014972	KP014564	KU670693	KU670768	-	-
Centropogon roseus Rusby	L. Lagomarsino 372 (GH)	Peru	KP014119	KP014827	KP014412	KP014628	KU670808	-	-
Centropogon salviaeformis Zahlb.	N. Muchhala 173	Ecuador	KP014230	KP014932	KP014554	KP014726	-	-	-
Centropogon simulans Lammers	L. Lagomarsino 402 (GH)	Peru	KP014281	KP014974	KP014461	KP014646	KU670770	-	-
Centropogon smithii E. Wimm.	L. Lagomarsino 68 (GH)	Costa Rica	KP014211	KP015008	KP014562	KP014772	-	-	-
Centropogon sodiroanus Zahlb.	N. Muchhala 106	Ecuador	KP014204	KP015011	KP014568	KU670706	-	-	-
Centropogon solanifolius Benth. 1	C. D. Bacon 261 (GB)	Colombia	KP014134	KP014839	KP014402	KU670696	-	-	-
Centropogon solanifolius Benth. 2	L. Lagomarsino 25 (GH)	Costa Rica	KP014132	KP014832	KP014398	KP014626	-	-	-
Centropogon solanifolius Benth. 3	D. Santamaría S-904 (GB)	Costa Rica	KP014133	KP014834	KP014397	KP014621	-	-	-
Centropogon subandinus Zahlb.	N. Muchhala 475	Ecuador	KP014207	KP014913	KP014569	KP014756	-	-	-

Centropogon talamancensis Wilbur	L. Lagomarsino 140 (GH)	Costa Rica	KP014302	KP014953	KP014467	KP014748	-	-	-
Centropogon tessmannii E. Wimm.	N. Muchhala 168	Ecuador	KP014150	KP014882	KP014344	KP014700	KU670773	-	-
Centropogon trachyanthus E. Wimm.	N. Muchhala 138	Ecuador	KP014137	KP014838	KP014401	KP014606	-	-	-
Centropogon trichodes E. Wimm.	L. Andersson 2546 (GB)	Ecuador	KP014213	KP014970	KP014535	KP014766	KU670780	DQ356217	DQ356098
Centropogon umbrosus E. Wimm	L. Lagomarsino 349 (GH)	Peru	KP014125	KP014825	-	KP014631	-	-	-
Centropogon unduavensis (Britton) Zahlb.	J. Solomon 15418 (LPB)	Bolivia	KP014290	KP014978	KP014453	-	-	-	-
Centropogon urubambae E. Wimm	L. Lagomarsino 347 (GH)	Peru	KP014148	KP014831	KP014414	KP014617	-	-	-
Centropogon valerioi Standley	D. Santamaría S-977 (GB)	Costa Rica	KP014215	KP014924	KP014545	KP014733	-	-	-
Centropogon vargasii B. A. Stein (ined.)	L. Lagomarsino 356 (GH)	Peru	KU670745	KU670681	KU670732		KU670772	-	-
Centropogon viriduliflorus Wimm	L. Lagomarsino 370 (GH)	Peru	KP014268	KP015006	KP014487	KP014665	-	-	-
Centropogon viriduliflorus Wimm	L. Valenzuela 712 (OSH)	Peru	KP014266	-	KP014448	-	-	-	-
Centropogon weberbaueri Zahlb.	L. Lagomarsino 317(GH)	Peru	KP014218	KP014934	KP014547	KP014736	-	-	-
Centropogon cf. *weberbaueri* Zahlb.	L. Lagomarsino 351 (GH)	Peru	KP014232	KP014935	KP014552	KP014735	-	-	-
Centropogon yarumalensis E. Wimm.	C. D. Bacon 263 (GB)	Colombia	KP014221	KP014918	KP014541	KP014725	KU670775	-	-
Centropogon yungasensis Britton	L. Lagomarsino 392 (GH)	Peru	KP014149	KP014818	KP014391	KP014619	KU670776	-	-
Centropogon sp. nov. (Lagom. & Santam. ined.)	L. Lagomarsino 354 (GH)	Peru	KP014269	KP014987	KP014489	KP014664	-	-	-
Centropogon sp.	A. Antonelli 611	Ecuador	KU670748	KU670687	KU670722	KU670705	-	-	-
Lobelia									
Lobelia bridgesii Hook & Arn.	M. Gustafsson 1019 (AAU)	Cultivated	KP014077	KP014800	KP014338	KP014584	-	-	-
Lobelia excelsa Bonpland	L. Lagomarsino 426 (GH); Lammers et al. 6357	Cultivated	KP014078	KP014801	KP014341	KP014586	DQ356146	DQ356212	-

Lobelia polyphylla Hook. & Arn.	Lammers et al. 6331 (GB)	Cultivated	-	-	-	-	DQ356123	DQ356177	-
Lobelia tupa L.	Cultivated, University of Aarhus; Lammers and Rodriguez 6329 (GB)	Cultivated	KP014076	KP014802	KP014339	KP014585	KU670784	DQ356211	-
Lysipomia									
Lysipomia cuspidata McVaugh	L. Andersson 2559 (GB)	Ecuador	KP014057	-	KP014318	KP014579	DQ356133	DQ356198	-
Lysipomia muscoides Hook f.	L. Lagomarsino 325 (GH)	Peru	KP014051	-	KP014312	KP014575	-	-	-
Lysipomia pumila (Wedd.) E. Wimm.	L. Lagomarsino 238 (GH)	Bolivia	KP014056	KP014791	KP014317	KP014580	KU670783	-	-
Lysipomia sphagnophila Griseb. Ex Wedd.	L. Andersson 2444 (GB)	Ecuador	KP014052	-	KP014313	KP014576	DQ356132	DQ356197	-
Lysipomia vitreola McVaugh	A. Antonelli 581 (GB)	Ecuador	KP014058	KP014792	KP014319	KU670704	-	-	-
***Siphocampylus* (excluding section *Hemisiphocampylus*)**									
Siphocampylus actinothrix E. Wimm.	L. Lagomarsino 403 (GH)	Peru	KP014086	KP014812	KP014367	KP014643	-	-	-
Siphocampylus aff. *actinothrix* E. Wimm.	L. Lagomarsino 361 (GH)	Peru	KP014086	KP014812	KP014367	KP014643	-	-	-
Siphocampylus affinis (Mirb.) McVaugh	B. Lojtnant 11426 (GB)	Ecuador	KP014270	-	KP014456	KP014661	DQ356155	DQ356223	DQ356104
Siphocampylus ambivalens Lammers	J. Wood 9986	Bolivia	KP014151	KP014806	KP014421	KP014650	-	-	-
Siphocampylus andinus Britton	L. Lagomarsino 226 (GH)	Bolivia	KP014260	KP014984	KP014500	KP014654	KU670782	-	-
Siphocampylus angustiflorus Schlechtend.	L. Lagomarsino 417 (GH)	Peru	KP014097	KP014887	KP014379	KP014633	KU670785	-	-
Siphocampylus antioquianus E. Wimm.	C. D. Bacon 242 (GB)	Colombia	KP014090	KP014943	KP014364	KU670710	-	-	-
Siphocampylus aureus Rusby	J. Wood 14956	Bolivia	KP014105	KP014898	KP014383	KU670718	KU670786	-	-
Siphocampylus ayersiae Lammers	L. Lagomarsino 212 (GH)	Bolivia	KP014297	KP014965	KP014437	KP014648	-	-	-
Siphocampylus betulaefolius (Cham.) G. Don.	S. Godoy 3000 (UEC)	Brazil	KP014153	KP014904	KP014420	KP014694	KU670788	-	-

Siphocampylus bilabiatus Zahlb.	A. Araujo-Murakami 4230 (MO)	Bolivia	KP014256	KP015016	KP014423	KP014677	KU670789	-	-
Siphocampylus boliviensis Zahlb.	L. Lagomarsino 239 (GH)	Bolivia	KP014252	KP014960	KP014483	KP014673	-	-	-
Siphocampylus brevicalyx E. Wimm.	N. Muchhala 480; Lojtnant et al 11826	Ecuador	KP014245	KP014901	KP014433	KP014715	KU670790	DQ356224	-
Siphocampylus chloroleucus E. Wimm	Asplund 13060 (S)	Ecuador	KP014098	KP014903	KP014377	KP014611	-	-	-
Siphocampylus citrinus E. Wimm	L. Lagomarsino 368 (GH)	Peru	KP014115	KP014896	KP014374	KP014604	KU670792	-	-
Siphocampylus clotho E. Wimm.	L. Lagomarsino 367 (GH)	Peru	KU670742	KU670686	KU670728	KU670714	KU670791	-	-
Siphocampylus convolvulaceus (Cham.) G. Don.	F. M. Souza 104 (UEC)	Brazil	KP014159	KP014907	KP014481	KP014645	-	-	-
Siphocampylus correoides Zahlb.	A. Timmermann 3 (AAU)	Bolivia	KP014298	KP014967	KP014436	KP014657	-	-	-
Siphocampylus corymbifer Pohl	A. Timmermann 9 (AAU)	Bolivia	KP014155	KP014941	KP014347	-	-	-	-
Siphocampylus corymbifer Pohl	A. Glaziou s.n.	Bolivia	KP014156	KP014940	KP014348	-	-	-	-
Siphocampylus corymbifer Pohl	L. Lagomarsino 216 (GH)	Bolivia	KP014157	KP014942	KP014346	KP014693	KU670796	-	-
Siphocampylus corynoides E. Wimm	L. Lagomarsino 353 (GH)	Peru	KP014117	KP014910	KP014361	KP014610	-	-	-
Siphocampylus dependens (Ruiz & Pavon) G. Don	L. Lagomarsino 316 (GH)	Peru	KP014261	KP015000	KP014485	KP014690	-	-	-
Siphocampylus elfriedii E. Wimm	L. Lagomarsino 387 (GH)	Peru	KP014084	KP014813	KP014371	KP014641	KU670793	-	-
Siphocampylus fiebrigii E. Wimm	S. Beck 31446 (LPB)	Bolivia	KP014100	KP014900	KP014382	KP014599	-	-	-
Siphocampylus flagelliformis Zahlb.	E. Garcia 2006 (GB)	Bolivia	KP014096	KP014883	KP014378	KP014612	-	-	-
Siphocampylus flagelliformis Zahlb.	L. Lagomarsino 220 (GH)	Bolivia	KP014099	KP014888	KP014380	KP014632	KU670794	-	-
Siphocampylus fulgens Hort.	Hatschbacch 44995 (GB)	Ecuador	KP014081	KP015014	KP014434	KP014719	EF141032	DQ356216	-
Siphocampylus giganteus (Cav.) G. Don.	L. Andersson 2432 (GB)	Ecuador	-	KP015002	KP014533	KP014761	DQ356154	DQ356222	DQ356103

Siphocampylus giganteus (Cav.) G. Don.	N. Muchhala 101	Ecuador	KP014205	KP015001	KP014534	KU670699	EF174621	-	-
Siphocampylus imbricatus (Cham.) G. Don.	P. Nuñéz 8182 (AAU)	Brazil	-	KU670679	KP014329	KU670698	-	-	-
Siphocampylus jelskii Zahlb.	L. Lagomarsino 321 (GH)	Peru	KP014199	KP014956	KP014567	KP014764	KU670795	-	-
Siphocampylus krauseanus E. Wimm.	L. Lagomarsino 418	Peru	-	KU670678	KU670736	KU670713	KU670797	-	-
Siphocampylus longipedunculatus Pohl	A. Antonelli 299 (GB)	Brazil	KP014238	KP014905	KP014471	KP014697	-	-	-
Siphocampylus lycioides (Cham.) G. Don.	R. Romero 3805 (UEC)	Brazil	KP014247	KP014808	KP014352	KP014710	KU670798	-	-
Siphocampylus macropodus (Billb.) G. Don.	A. Antonelli 298 (GB)	Brazil	KP014107	KP014890	KP014365	KP014639	DQ356153	DQ356221	DQ356102
Siphocampylus matthiaei A. DC.	L. Lagomarsino 313 (GH)	Peru	KP014190	KP014968	KP014536	KP014765	KU670807	-	-
Siphocampylus membraceus Britton	A. Gentry 44,526 (MO)	Bolivia	KP014094	KP014885	KP014388	-	-	-	-
Siphocampylus nematosepalus (Donn. Smith) E. Wimm	L. Lagomarsino 164 (GH)	Costa Rica	KP014300	KP014952	KP014463	KP014741	KU670800	-	-
Siphocampylus nemoralis Griseb.	D. Spooner 6605	Bolivia	KP014112	KP014944	KP014426	-	-	-	-
Siphocampylus neurotrichus E. Wimm	M. Nee 42251 (NY)	Bolivia	KP014108	KP014889	KP014381	KP014603	-	-	-
Siphocampylus nitidus Pohl	J. Pirani 689 (UEC)	Brazil	-	-	KP014349	-	-	-	-
Siphocampylus oblongifolius Rusby	J. L. Clark 6687 (OSH)	Bolivia	KP014093	KP014884	KP014358	KP014636	-	-	-
Siphocampylus oblongifolius Rusby	S. Beck 30201 (LPB)	Bolivia	KP014095	KP014886	KP014389	-	-	-	-
Siphocampylus obovatus (G. Don) E. Wimm	L. Lagomarsino 350 (GH)	Peru	KP014116	-	KP014360	KP014609	-	-	-
Siphocampylus orbignianus A. DC.	L. Lagomarsino 217 (GH)	Bolivia	KP014152	KP014807	KP014422	KP014655	-	-	-
Siphocampylus puberulus E. Wimm	Vargas 226 (LPB)	Bolivia	KP014109	KP014964	KP014427	-	-	-	-
Siphocampylus pubescens Benth.	B. Ollgaard 74216 (GH)	Ecuador	KP014080	-	KP014419	-	-	-	-
Siphocampylus aff. *rictus* Lammers	L. Lagomarsino 341 (GH)	Peru	KP014082	KP014811	KP014363	KP014640	-	-	-

Siphocampylus rosmarinifolius G. Don.	L. Lagomarsino 331 (GH)	Peru	KP014113	KP014909	KP014362	KP014634	KU670801	-	-
Siphocampylus rusbyanus Britton	A. Fuentes 4760 (MO)	Bolivia	KP014264	KP015012	KP014431	KP014689	KU670802	-	-
Siphocampylus scandens (HBK) G. Don.	L. Andersson 2515 (GB)	Ecuador	KP014248	KP014973	KP014356	KP014592	DQ356150	DQ356218	DQ356099
Siphocampylus sceptrum Decaisne apud Linden	L. Dorr 20513	Venezuela	KP014242	KP015009	KP014392	KP014597	-	-	-
Siphocampylus smilax Lammers	L. Lagomarsino 250 (GH)	Bolivia	KP014079	KP014908	KP014342	KP014649	KU670803	-	-
Siphocampylus sparsipilus E. Wimm	L. Lagomarsino 197 (GH)	Bolivia	KP014278	KP014939	KP014492	KP014652	KU670804	-	-
Siphocampylus tunarensis Zahlb.	L. Lagomarsino 232 (GH)	Bolivia	KP014271	KP014975	KP014435	KP014674	KU670752	-	-
Siphocampylus tunicatus Zahlb.	L. Lagomarsino 235 (GH)	Bolivia	KP014272	KP014991	KP014443	KP014676	KU670754	-	-
Siphocampylus tupaeformis Zahlb.	A. Timmermann 7 (LPB)	Bolivia	KP014111	KP014962	KP014429	KP014708	-	-	-
Siphocampylus umbellatus (HBK) G. Don.	L. Lagomarsino 193 (GH)	Bolivia	KP014275	KP014994	KP014442	KP014672	-	-	-
Siphocampylus vatkeanus Zahlb.	N. Paniagua 5774 (MO)	Bolivia	KP014088	KP014816	KP014369	KP014608	KU670753	-	-
Siphocampylus veteranus Wimm	L. Lagomarsino 388 (GH)	Peru	KP014087	KP014815	KP014373	KP014644	KU670781	-	-
Siphocampylus virgatus A. DC.	L. Lagomarsino 343 (GH)	Peru	KP014091	KP014958	KP014357	KP014607	KU670755	-	-
Siphocampylus werdermannii E. Wimm	L. Lagomarsino 245 (GH)	Bolivia	KP014106	KP014893	KP014376	KP014602	-	-	-
Siphocampylus westinianus (Billb.) Pohl	A. Antonelli 308 (GB)	Brazil	KP014154	KP014906	KP014472	KP014587	-	-	-
Siphocampylus sp.	L. Lagomarsino 333 (GH)	Peru	KP014262	KP015010	KP014486	KP014691	-	-	-
Siphocampylus sp. nov. 1 (Lagom. & Santam. ined.)	L. Lagomarsino 241 (GH)	Bolivia	KP014274	KP014990	KP014440	KP014675	-	-	-
Siphocampylus sp. nov. 2 (Lagom. & Santam. ined.)	L. Lagomarsino 400 (GH)	Peru	KP014085	KP014814	KP014372	KP014642	-	-	-

OUTGROUP

Apetahia									
Apetahia margaretae (F. Br.) E. Wimm.	N/A	French Polynesia	-	-	-	-	DQ285286	DQ285169	-
Brighamia									
Brighamia insignis A. Gray	Antonelli 251 (GB)	Hawaii	-	-	-	-	AF042664	DQ356189	-
Campanula									
Campanula asperuloides Harms	Antonelli 283 (GB)	Greece	-	-	-	-	DQ356117	DQ356170	DQ356090
Campanula carpatica Jacq.	Gaskin 121 (MO)	unknown	-	-	-	-	EU713410	-	-
Campanula latifolia L.	Antonelli 252(GB)	Sweden	-	-	-	-	EF141027	DQ356169	DQ356089
Campanula medium L.	L. Lagomarsino 422 (GH)	USA	KP014048	-	KP014307	KP014571	-	-	-
Campanula pyramidalis Gilib.	Eddie 96089 (EGHB)	unknown	-	-	-	-	EU713429	GQ254919	-
Campanula trachelium Brot.	Antonelli 281 (GB)	Sweden	-	-	-	-	DQ356118	DQ356171	DQ356091
Canarina									
Canarina canariensis (L.) Vatke	Eddie 96048 (EGHB); Andersson 2394 (GB)	Spain	-	-	-	-	EU713353	DQ356167	DQ356087
Clermontia									
Clermontia arborescens (H. Mann) Hillebr.	I. Carter 1	Hawaii	-	-	KU670724	KU670694	KU670779	DQ285141	-
Clermontia fauriei H. Lév.	Johansen 1	Hawaii	-	-	-	-	JX500350	DQ285142	-
Clermontia kakeana Meyen.	Johansen 5; Takeuchi Koolau 56a (GB)	Hawaii	-	-	-	-	JX500348	DQ285143	DQ356092
Cyanea									
Cyanea angustifolia Hillebr.	Takeuchi et al. 1802 (GB)	Hawaii	-	-	-	-	DQ356119	DQ356173	-
Cyanea asplenifolia (H. Mann) Hillebr.	N/A	Hawaii	-	-	-	-	JX500351	-	-
Cyanea koolauensis Lammers, Givnish & Systma	Takeuchi & Pyle 2284 (GB)	Hawaii	-	-	-	-	DQ356128	DQ356193	-
Delissea									
Delissea undulata Gaudich.	Skottsberg 691 (GB)	Hawaii	-	-	-	-	-	DQ356188	-
Diastatea									

Diastatea micrantha (Kunth) McVaugh	L. Lagomarsino 44; Lojtnant and Molau 15184	Costa Rica	KP014066	KP014803	KP014327	KP014581	DQ356138	DQ356203	DQ356095
Downingia									
Downingia bacigalupii Weiler	Mason 14384(S)	California	-	-	-	-	EF141031	DQ356183	-
Downingia insignis Greene	Tiehm 12143 (S)	California	-	-	-	-	EF141030	DQ356185	-
Grammatotheca									
Grammatotheca bergiana (Cham.) C. Presl	Bean & Viviers 2628 (GB)	South Africa	-	-	-	-	DQ356116	DQ356168	DQ356088
Hippobroma									
Hippobroma longiflora (L.) G. Don	L. Lagomarsino 346; Andersson and Nilsson 2492 (GB)	Peru	KP014072	-	KP014337	KP014782	KU670778	DQ356206	DQ356096
Isotoma									
Isotoma fluviatilis F. Muell. Ex Bentham	Cultivated, University of Aarhus, no voucher	Clutivated	KP014054	KP014790	KP014321	KP014783	DQ356161	DQ356230	DQ356108
Jasione									
Jasione montana L.	F.I.Sales & C.Hedge 9898 (EGHB); Andersson 2562 (GB)	unknown	-	-	-	-	EU713354	DQ356174	-
Legousia									
Legousia hybrid Delarb.	Corneliuson s.n.; (GB 152920)	Sweden	-	-	-	-	DQ356163	DQ356234	DQ356111
Lobelia									
Lobelia aguana E. Wimm.	Koch 82120 (NY)	Mexico	-	-	-	-	DQ356122	DQ356176	-
Lobelia alsinoides Lam.	Larsen 34131 (AAU)	Thailand	KP014050	KP014785	KP014309	KP014572	-	-	
Lobelia anceps L.	Skottsberg 4536 (GB)	Chile	-	-	-	-	DQ356124	DQ356184	-
Lobelia aquatica Cham.	Jacobs et al. 4080(GB)	Guyana	-	-	-	-	EF141029	DQ356182	-
Lobelia assurgens var. *portoricensis* Urb.	Trejo 28 (GH)	Puerto Rico	-	-	KU670730	-	-	-	-
Lobelia cardinalis L.	L. Lagomarsino 423 (GH); T. Lammers 8710 (F)	USA	KP014060	KP014794	KP014314	KU670717	AY655144	DQ356231 (Vincent 4377 (GB))	AF130187

Lobelia cardinalis subsp. *graminea* (Lam.) McVaugh	R. Kriebel 5570 (NY)	Guatemala	KP014061	KP014795	KP014315	-	-	-	-
Lobelia chinensis Hance	G.Kokubugata11397; Hashimoto 409 (GB)	Japan	-	-	-	-	AB645974	DQ356228	-
Lobelia cliffortiana L.	R. A. Howard 15704 (NY)	Puerto Rico	-	KU670691	KU670733	-	-	-	-
Lobelia cordifolia Hook. & Arn.	Mexia 8780 (S)	Mexico	-	-	-	-	-	DQ356204	-
Lobelia coronopifolia L.	Dahlstrand 1084(GB)	South Africa	-	-	-	-	EF141025	DQ356181	-
Lobelia digitalifolia (Griseb.) Urb.	R. A. Howard 19407 (NY)	Guadeloupe	-	-	KP014324	-	-	-	-
Lobelia dortmanna L.	Andersson 2561 (GB)	Sweden	-	-	-	-	DQ356162	DQ356232	DQ356109
Lobelia erinus L.	Andersson 2570 (GB)	Cultivated	-	-	-	-	HM850129	DQ356233	DQ356110
Lobelia exaltata Pohl	Antonelli 335 (GB)	Brazil	-	-	-	-	DQ356135	DQ356200	DQ356093
Lobelia fistulosa Raf.	Antonelli & Andersson 279 (GB)	Brazil	-	-	-	-	DQ356136	DQ356201	DQ356094
Lobelia gibberoa Hemsl.	Jaasund s.n.; (GB 1.1.67)	Kenya	-	-	-	-	DQ356127	DQ356192	-
Lobelia graniticola E. Wimm.	Thulin & Mhoro 3210 (C)	Tanzania	-	-	-	-	DQ356129	DQ356194	-
Lobelia gregoriana Baker f.	Hedberg 1608 (S)	Kenya	-	-	-	-	DQ356187	DQ356187	-
Lobelia hederacea Cham.	A. Schinini 12141 (AAU)	Argentina	KP014053	KP014797	KP014323	KP014578	-	-	-
Lobelia hypoleuca Hillebr.	Selling & Skottsberg 3194 (GB)	Hawaii	-	-	-	-	DQ356126	DQ356191	-
Lobelia inflata L.	H. Schmidt 1531 (MO)	USA	KP014062	-	KP014320	-	-	-	-
Lobelia irasuensis Planh. & Oerst.	L. Lagomarsino 5 (GH); Burger & Burger 8151 (GB)	Costa Rica	-	KU670690	KU670720	-	KU670750	DQ356175	-
Lobelia kalmii L.	L. Lagomarsino 424 (GH); D. Boufford 7292	USA	KP014059	KP014793	KP014316	KP014577	DQ356166 (Boufford 7292)	EF126736	DQ356114
Lobelia kraussii Graham	Hill 25677(NY)	Dominica	-	-	-	-	EF141024	DQ356179	-

Lobelia laxiflora Kunth.	L. Lagomarsino 289 (GH); D. Santamaria S-985	Panama	KP014065	KP014799	KP014326	KP014777	DQ356143	DQ356209	-
Lobelia leschenaultiana Skottsb.	Skottsberg s.n.; (GB 11.XII.1926)	Sri Lanka	-	-	-	-	DQ356131	DQ356196	-
Lobelia martagon Hitchc.	W. Judd 5375 (NY)	Jamaica	KP014073	-	KP014335	-	KU670806	DQ356205	-
Lobelia nana Kunth.	L. Lagomarsino 228 (GH)	Bolivia	KP014063	KP014796	KP014328	KP014775	-	-	-
Lobelia oligophylla (Wedd.) Lammers	C. M. Taylor 11210 (NY); (Harling et al. 6807)	Argentina	KP014055	-	KP014322	KP014784	KU670787	DQ356227	-
Lobelia persicifolia Cav.	Whitefand 3782 (GH)	Guadeloupe	-	-	KU670731	-	-	-	-
Lobelia physaloides A. Cunn.	Cameron s.n. (AAU)	New Zealand	-	-	KP014311	KP014574	-	-	-
Lobelia portoricensis (Vatke) Urb.	Boom 10069 (NY)	Puerto Rico	-	-	-	-	DQ356142	DQ356208	-
Lobelia roughii Hook f.	Skottsberg s.n. (GB)	New Zealand	-	-	-	-	DQ356165	EF126737	DQ356113
Lobelia sonderiana (Kuntze) Lammers	Volk 1095 (S)	Namibia	-	-	-	-	DQ356130	DQ356195	-
Lobelia stricklandiae Gilliland	Fisher & Thweicher 291 (S)	Zimbabwe	-	-	-	-	-	DQ356186	-
Lobelia stricta M. Martens & Galeotti	Wilbur et al. 7849 (NY)	Dominica	-	-	KU670738	-	DQ356141	DQ356207	-
Lobelia tomentosa L. f.	Dahlstrand 2600(GB)	South Africa	-	-	-	-	EF141028	DQ356180	-
Lobelia viridiflora McVaugh	Weaver 910 (GH)	Jamaica	-	-	KU670735	-	-	-	-
Lobelia xalapensis Kunth	Santamaria S-1009 (GB)	Costa Rica	-	-	-	-	DQ356144	DQ356210	-
Lobelia yuccoides Hillebr.	Cranwell et al. 2909 (GB)	Hawaii	-	-	-	-	DQ356125	DQ356190	-
Lobelia zeylanica L.	D. Boufford 24898 (GH)	Taiwan	-	-	KP014310	KP014573	-	-	-
Pratia									
Pratia nummularia (Lam.) A. Braun & Asch.	Andersson 2571 (GB)	Cultivated	-	-	-	-	DQ356164	DQ356235	DQ356112
Pratia purpurascens E. Wimm.	Egerod s.n. (GB)	Australia	-	-	-	-	DQ356160	DQ356229	DQ356107

Siphocampylus* section *Hemisiphocampylus										
Siphocampylus decumbens (Rich.) Juss & A. DC.	P. Acevedo-Rodriguez 14172 (NY)	Dominican Republic	KP014067	-	KP014330	-	-	-	-
Siphocampylus domingensis A. DC.	A. Veloz 2001 (JBSD)	Dominican Republic	KP014068	KP014787	KP014331	KP014582	-	-	-
Siphocampylus igneus Urb.	J. R. Abbott 21003 (FLAS)	Dominican Republic	KP014069	KP014788	KP014332	KP014779	-	-	-
Siphocampylus manettiaeflorus Hook.	Cultivated, Marbury Bot. Garden, no voucher	Cultivated	KP014074	KP014786	KP014334	KP014780	KU670799	-	-
Siphocampylus sonchifolius (Sw.) McVaugh	T. Clase 3048 (JBSD)	Dominican Republic	KP014070	KP014789	KP014333	KP014778	-	-	-
Solenopsis									
Solenopsis laurentia (L.) C. Presl.	Dept.Syst.Bot. 381 (GB)	Cultivated	-	-	-	-	DQ356134	DQ356199	-
Trematolobelia									
Trematolobelia macrostachys (Hook. & Arn.) Zahlbr.	Fagerlind 6872 (S)	Hawaii	-	-	-	-	DQ356137	DQ356202	-
Triodanis									
Triodanis perfoliata (L.) Nieuwl.	L. Lagomarsino 425 (GH)	USA	KP014049	-	KP014308	-	-	-	-

Herbarium acronyms follow the Index Herbariorum. Taxa are organized alphabetically within the ingroup and outgroup categories

Table S2. Divergence time estimation to assess sensitivity to dating method, calibration selection, and branch length heterogeneity.

	Campanulaceae	Campanuloideae	Lobelioideae	Neotropical Lobelioideae	*Lobelia* section *Tupa*	*Lysipomia*	centropogonids
**treePL dates from RAxML bootstrap trees**							
All priors	58.19 (55.89-61.62)	56.13 (55.87-57.0)	54.98 (51.71-56.00)	20.15 (18.06-22.04)	1.07 (0.36-1.35)	13.05 (11.13-14.36)	5.12 (4.24-5.83)
Three priors (fossil and secondary ages)	57.15 (55.90–61.76)	55.93 (55.87–56.0)	55.03 (51.72–56.0)	20.24 (18.09–22.04)	0.86 (0.34–1.33)	13.08 (11.13–14.37)	5.32 (4.22–5.86)
Two priors (fossil and root age)	66.89 (66.88–67.0)	65.41 (60.64–66.95)	64.65 (56.13–66.94)	24.73 (20.74–25.40)	1.01 (0.15–1.20)	15.21 (13.01–16.38)	6.22 (4.76–6.67)
One prior (root age only)	66.69 (66.88–67.0)	65.34 (60.65–66.96)	63. 66 (56.13–66.93)	23.58 (21.19–25.25)	1.01 (0.147–1.20)	15.3 (12.85–16.38)	6.24 (4.76–6.68)
All priors, *Lysipomia* removed	60.17 (55.98–62.09)	56 (55.87–56.0)	53.42 (50.96–56.12)	17.33 (13.97–18.31)	0.34 (0.31–1.31)	N/A	4.62 (4.14–5.45)
**BEAST dates, all with expanded outgroup**							
All priors, exponential relaxed clock	57.1 (44-69.55)	46.57 (36.28-56.39)	49.8 (35.88-65.20)	16.26 (9.44-22.51)	0.9 (0.18-2.63)	8.2 (4.70-13.06)	5.94 (3.32-9.54)
**All priors, lognormal relaxed clock***	**58.78 (49.87-68.04)**	**53.48 (45.60-62.11)**	**46.79 (39.07-55.07)**	**17.74 (14.32-20.32)**	**1.35 (0.42-2.78)**	**11.69 (9.00-15.24)**	**4.98 (3.95-6.13)**
Three priors (fossil and secondary ages), lognormal relaxed clock	59.2 (46.81-72.03)	48.55 (38.38-59.31)	51.78 (37.87-66.55)	16.07 (10.54-22.32)	0.95 (0.22-2.75)	8.97 (5.08-13.83)	6.55 (3.74-9.95)
Two priors (fossil and root age), lognormal relaxed clock	62.47 (48.31-76.38)	53.23 (39.67-68.62)	51.52 (34.62-69.17)	17.78 (9.69-24.49)	0.97 (0.30-2.90)	9.24 (5.03-13.89)	6.71 (3.87-10.46)
One prior (root age only), lognormal relaxed clock	53.4 (35.61-69.51)	20.33 (9.69-34.34)	43.62 (19.68-57.10)	12.11 (6.27-18.80)	0.68 (0.10-2.17)	6.37 (2.84-9.89)	4.77 (2.25-7.67)
All priors, *Lysipomia* removed	58.77 (47.0-71.94)	49.16 (37.82-59.07)	50.05 (35.34-66.99)	11.8 (6.93-17.63)	0.85 (0.15-0.86)	N/A	6.11 (3.78-9.47)

Analysis in boldface denoted with an asterisk is the one presented in the main text and used in subsequent diversification analyses. Age estimates reported for major clades relevant to the study reported in million years (Ma). Root node = crown group Campanulaceae.

Table S3. Trait codings for the four traits used in our BiSSE analyses.

	Andean Distribution	Elevation	Fruit	Pollinator
Burmeistera almedae Wilbur	0	1	1	1
Burmeistera aff. *bullatifolia* J. Garzón & F. Gonzalez	1	1	1	1
Burmeistera aff. *bullatifoli*a J. Garzón & F. Gonzalez	1	1	1	1
Burmeistera ceratocarpa Zahlb.	1	0	1	1
Burmeistera chiriquiensis Wilbur	0	1	1	1
Burmeistera crebra McVaugh	0	1	1	1
Burmeistera cyclostigmata J. D. Smith	0	1	1	1
Burmeistera dendrophila E. Wimm	0	1	1	1
Burmeistera domingensis Jeppesen	1	0	1	1
Burmeistera fuchsioides J. Garzón & F. Gonzalez	1	1	1	1
Burmeistera mcvaughii Wilbur	0	1	1	1
Burmeistera mcvaughii Wilbur	0	1	1	1
Burmeistera microphylla J. D. Smith	0	0	1	1
Burmeistera aff. *minutiflora* J. Garzón & F. Gonzalez	1	1	1	0
Burmeistera morii Wilbur	0	0	1	1
Burmeistera multiflora Zahlb.	1	0	1	1
Burmeistera obtusifolia E. Wimm	0	1	1	1
Burmeistera obtusifolia E. Wimm	0	1	1	1
Burmeistera parviflora E. Wimm	0	1	1	0
Burmeistera racemiflora Lammers	1	0	1	1
Burmeistera refracta E. Wimm.	1	0	1	1
Burmeistera refracta E. Wimm.	1	0	1	1
Burmeistera sodiroana Zahlb.	1	1	1	1
Burmeistera succulenta H. Karst. & Triana	1	0	1	1
Burmeistera tenuiflora J.D. Smith	0	0	1	1
Burmeistera toroensis Wilbur	0	0	1	1
Burmeistera truncata Zahlbr.	1	0	1	1
Burmeistera utleyi Wilbur	0	1	1	1
Burmeistera variabilis (Gleason) E. Wimm.	1	0	1	1
Burmeistera vulgaris E. Wimm.	0	0	1	1
Burmeistera zurquiensis Wilbur	0	1	1	1
Burmeistera sp.	1	1	1	1
Burmeistera sp. nov.	0	1	1	0

Centropogon aequatorialis E. Wimm.	1	1	1	1
Centropogon argutus E. Wimm.	1	1	1	1
Centropogon asclepiadeus (Willd.) E. Wimm.	1	1	1	1
Centropogon azuayensis Jeppesen	1	1	1	1
Centropogon baezanus Jeppesen	1	1	1	1
Centropogon bangii Zahlb.	1	0	1	1
Centropogon brittonianus Zahlb.	1	1	1	1
Centropogon coccineus (Hook.) Regel ex B.D. Jacks.	0	0	1	1
Centropogon comosus Gleason	1	1	1	1
Centropogon congestus Gleason	0	0	1	1
Centropogon cordifolius Benth.	0	0	1	1
Centropogon cornutus (L.) Druce	0	0	1	1
Centropogon costaricae (Vatke) E. Wimm.	0	1	1	1
Centropogon dianae Lammers	1	1	1	1
Centropogon dissectus E. Wimm.	1	1	1	1
Centropogon dombeyanus E. Wimm	1	1	1	1
Centropogon eilersii Lammers & M.O. Dillon	1	1	1	1
Centropogon erythraeus Drake	1	1	1	1
Centropogon featherstonei Gleason	1	1	1	1
Centropogon ferrugineus (L.f) Gleason	0	1	1	1
Centropogon ferrugineus (L.f) Gleason	0	1	1	1
Centropogon ferrugineus (L.f) Gleason	0	1	1	1
Centropogon foetidus (Kunth) E. Wimm.	1	1	1	1
Centropogon gamosepalus Zahlb.	1	0	1	1
Centropogon glabrifilis (E. Wimm.) Jeppsen	1	1	1	1
Centropogon gloriosus (Britton) Zahlb.	1	1	1	1
Centropogon grandidentatus (Schltdl.) Zahlbr.	0	0	1	1
Centropogon granulosus subsp. *granulosus* Stein (ined.) 1	1	0	1	1
Centropogon granulosus subsp. *granulosus* Stein (ined.) 2	1	0	1	1
Centropogon granulosus subsp. *lugens* Stein (ined)	1	0	1	1
Centropogon granulosus subsp. *nutans* Stein (ined.)	0	0	1	1
Centropogon granulosus subsp. *tortilis* Stein (ined.)	0	0	1	1
Centropogon granulosus C. Presl. 1	1	0	1	1
Centropogon granulosus C. Presl. 2	0	0	1	1

Centropogon granulosus C. Presl. 3	0	0	1	1
Centropogon granulosus C. Presl. 4	0	0	1	1
Centropogon gutierrezii (Planch & Oerst.) E. Wimm.	0	1	1	1
Centropogon hirtus (Cav.) C. Presl.	1	1	1	1
Centropogon incanus (Britton) Zahlb.	1	1	1	1
Centropogon incanus (Britton) Zahlbr..	1	1	1	1
Centropogon isabellinus E. Wimm.	1	1	1	1
Centropogon leucocarpus McVaugh	0	1	1	1
Centropogon llanganatensis Jeppsen	1	1	1	1
Centropogon luteus E. Wimm.	1	1	1	1
Centropogon luteynii Wilbur	0	1	1	1
Centropogon macbridei Gleason	1	1	1	1
Centropogon macrocarpus Zahlb.	1	1	1	1
Centropogon macrophyllus (G. Don.) E. Wimm	1	0	1	1
Centropogon magnificus Zahlb.	1	1	1	1
Centropogon mandonis Zahlb.	1	1	1	1
Centropogon mandonis Zahlb.	1	1	1	1
Centropogon medusa E. Wimm.	1	1	1	1
Centropogon minimus McVaugh	1	1	1	1
Centropogon nervosus Wimm	1	1	1	1
Centropogon nigricans Zahlb.	1	1	1	1
Centropogon nigricans Zahlb.	1	1	1	1
Centropogon palmanus (J. D. Smith) E. Wimm.	0	0	1	1
Centropogon panamensis Wilbur	0	0	1	1
Centropogon perlongus Gleason	1	1	1	1
Centropogon peruvianus (E. Wimm.) McVaugh	1	1	1	1
Centropogon pichinchensis Zahlb.	1	1	1	1
Centropogon preslii E. Wimm.	1	1	1	1
Centropogon pulcher Zahlb.	1	0	1	1
Centropogon reticulatus Drake	1	1	1	1
Centropogon rex E. Wimm.	1	1	1	1
Centropogon roseus Rusby	1	0	1	1
Centropogon salviaeformis Zahlb.	1	1	1	1
Centropogon simulans Lammers	1	1	1	1
Centropogon smithii E. Wimm.	0	1	1	1
Centropogon sodiroanus Zahlb.	1	1	1	1

Centropogon solanifolius Benth. 1	1	1	1	1
Centropogon solanifolius Benth. 2	0	1	1	1
Centropogon solanifolius Benth. 3	0	1	1	1
Centropogon subandinus Zahlb.	1	1	1	1
Centropogon talamancensis Wilbur	0	1	1	1
Centropogon tessmannii E. Wimm.	1	0	1	1
Centropogon trachyanthus E. Wimm.	1	1	1	1
Centropogon trichodes E. Wimm.	1	1	1	1
Centropogon umbrosus E. Wimm	1	1	1	1
Centropogon unduavensis (Britton) Zahlb.	1	1	1	1
Centropogon urubambae E. Wimm	1	0	1	1
Centropogon valerii Standley	0	1	1	1
Centropogon vargasii B. A. Stein (ined.)	1	1	1	1
Centropogon viriduliflorus Wimm	1	1	1	1
Centropogon viriduliflorus Wimm	1	1	1	1
Centropogon weberbaueri Zahlb.	1	1	1	1
Centropogon cf. *weberbaueri* Zahlb.	1	1	1	1
Centropogon yarumalensis E. Wimm.	1	1	1	1
Centropogon yungasensis Britton	1	1	1	1
Centropogon sp. nov. (Lagom. & Santam. ined.)	1	1	1	1
Centropogon sp.	1	1	1	1
Lobelia bridgesii Hook & Arn.	1	0	0	1
Lobelia excelsa Bonpland	1	0	0	1
Lobelia polyphylla Hook. & Arn.	1	0	0	1
Lobelia tupa L.	1	0	0	1
Lysipomia cuspidata McVaugh	1	1	0	0
Lysipomia muscoides Hook f.	1	1	0	0
Lysipomia pumila (Wedd.) E. Wimm.	1	1	0	0
Lysipomia sphagnophila Griseb. Ex Wedd.	1	1	0	0
Lysipomia vitreola McVaugh	1	1	0	0
Siphocampylus actinothrix E. Wimm.	1	1	0	1
Siphocampylus aff. *actinothrix* E. Wimm.	1	1	0	1
Siphocampylus affinis (Mirb.) McVaugh	1	1	0	1
Siphocampylus ambivalens Lammers	1	1	0	1
Siphocampylus andinus Britton	1	1	0	1
Siphocampylus angustiflorus Schlechtend.	1	1	0	1

Siphocampylus antioquianus E. Wimm.	1	1	0	1
Siphocampylus aureus Rusby	1	0	0	1
Siphocampylus ayersiae Lammers	1	1	0	1
Siphocampylus betulaefolius (Cham.) G. Don.	0	0	0	1
Siphocampylus bilabiatus Zahlb.	1	0	0	1
Siphocampylus boliviensis Zahlb.	1	1	0	1
Siphocampylus brevicalyx E. Wimm.	1	1	0	1
Siphocampylus chloroleucus E. Wimm	1	1	0	1
Siphocampylus citrinus E. Wimm	1	1	0	1
Siphocampylus clotho E. Wimm.	1	1	0	1
Siphocampylus convolvulaceus (Cham.) G. Don.	0	0	0	1
Siphocampylus correoides Zahlb.	1	1	0	1
Siphocampylus corymbifer Pohl	1	0	0	1
Siphocampylus corymbifer Pohl	1	0	0	1
Siphocampylus corymbifer Pohl	1	0	0	1
Siphocampylus corynoides E. Wimm	1	1	0	1
Siphocampylus dependens (Ruiz & Pavon) G. Don	1	1	0	1
Siphocampylus elfriedii E. Wimm	1	1	0	1
Siphocampylus fiebrigii E. Wimm	1	0	0	1
Siphocampylus flagelliformis Zahlb.	1	1	0	1
Siphocampylus flagelliformis Zahlb.	1	1	0	1
Siphocampylus fulgens Hort.	0	0	0	1
Siphocampylus giganteus (Cav.) G. Don.	1	1	0	1
Siphocampylus giganteus (Cav.) G. Don.	1	1	0	1
Siphocampylus imbricatus (Cham.) G. Don.	0	0	0	1
Siphocampylus jelskii Zahlb.	1	1	0	1
Siphocampylus krauseanus E. Wimm.	1	1	0	1
Siphocampylus longipedunculatus Pohl	0	0	0	1
Siphocampylus lycioides (Cham.) G. Don.	0	0	0	1
Siphocampylus macropodus (Billb.) G. Don.	0	0	0	1
Siphocampylus matthiaei A. DC.	1	1	0	1
Siphocampylus membraceus Britton	1	0	0	1
Siphocampylus nematosepalus (Donn. Smith) E. Wimm	0	1	0	1
Siphocampylus nemoralis Griseb.	1	1	0	1
Siphocampylus neurotrichus E. Wimm	1	0	0	1

Siphocampylus nitidus Pohl	0	0	0	1
Siphocampylus oblongifolius Rusby	1	0	0	1
Siphocampylus oblongifolius Rusby	1	0	0	1
Siphocampylus obovatus (G. Don) E. Wimm	1	1	0	1
Siphocampylus orbignianus A. DC.	1	0	0	1
Siphocampylus puberulus E. Wimm	1	1	0	1
Siphocampylus pubescens Benth.	1	1	0	1
Siphocampylus aff. *rictus* Lammers	1	1	0	1
Siphocampylus rosmarinifolius G. Don.	1	1	0	1
Siphocampylus rusbyanus Britton	1	1	0	1
Siphocampylus scandens (HBK) G. Don.	1	1	0	1
Siphocampylus sceptrum Decaisne apud Linden	1	1	0	1
Siphocampylus smilax Lammers	1	0	0	1
Siphocampylus sparsipilus E. Wimm	1	1	0	1
Siphocampylus tunarensis Zahlb.	1	1	0	1
Siphocampylus tunicatus Zahlb.	1	1	0	1
Siphocampylus tupaeformis Zahlb.	1	1	0	1
Siphocampylus umbellatus (HBK) G. Don.	1	1	0	1
Siphocampylus vatkeanus Zahlb.	1	1	0	1
Siphocampylus veteranus Wimm	1	1	0	1
Siphocampylus virgatus A. DC.	1	0	0	1
Siphocampylus werdermannii E. Wimm	1	1	0	1
Siphocampylus westinianus (Billb.) Pohl	0	0	0	1
Siphocampylus sp.	1	1	0	1
Siphocampylus sp. nov. 1 (Lagom. & Santam. ined.)	1	1	0	1
Siphocampylus sp. nov. 2 (Lagom. & Santam. ined.)	1	1	0	1

Character States are as follows: Andean Distribution— 0= extra-Andean, 1= Andean; Elevation— 0= Low elevation (<1900m), 1= High elevation (>1900 m); Fruit— 0= Capsule, 1= Berry; Pollinator— 0– Invertebrate, 1– Vertebrate

Table S4. Paleoenvironmental-dependent diversification analyses using paleoaltimetry (a) and Cenozoic climate data (b).

a) Paleoaltitude-dependence diversification models

Models	Mode of dependency	NP	logL	AICc	ΔAIC	*P* (LRT)	λ	α	μ	β
λ constant (no μ) (Yule)	-	1	-313.082	628.184	89.408	<0.001	1.016	-	-	-
λ and μ constant	-	2	-276.699	557.458	18.682	<0.001	2.915	-		-
**λ Alti. and no μ**	**Exponential**	**2**	**-267.358**	**538.776**	**0**	**Best**	**0.0010**	**0.00249**	**-**	**-**
λ Alti. and μ constant	Exponential	3	-266.863	539.848	1.072	0.3197	0.0004	0.00258	0.0184	-
λ constant and μ Alti.	Exponential	3	-271.228	548.578	9.802	0.999	2.3894	-	6.1267	-0.00026
λ Alti. and μ Alti.	Exponential	4	-266.523	541.250	2.474	0.4339	0.0016	0.00213	0.0611	-0.03312
λ Alti. and no μ	Linear	2	-281.176	566.413	27.637	0.999	2.1972	-0.00077	-	-
λ Alti. and μ constant	Linear	3	-268.544	543.211	4.435	0.999	2.3254	-0.00094	0.9437	-
λ constant and μ Alti.	Linear	3	-271.499	549.120	10.344	0.999	2.1654	-	4.1010	-0.00065
λ Alti. and μ Alti.	Linear	4	-270.284	548.773	9.997	0.999	2.7205	0.00011	1.7161	-0.00013

b) Temperature-dependence diversification models

**Models**	**Mode of dependency**	**NP**	**logL**	**AICc**	**ΔAIC**	***P* (LRT)**	**λ**	**α**	**μ**	**β**
λ constant (no μ) (Yule)	-	1	-313.082	628.184	92.14	<0.001	1.016	-	-	-
λ and μ constant	-	2	-276.699	557.458	21.414	<0.001	2.915	-		-
λ Temp. and no μ	Exponential	2	-268.613	541.286	5.242	0.999	2.7463	-0.43320	-	-
λ Temp. and μ constant	Exponential	3	-265.353	536.829	0.785	0.999	2.5085	-0.14360	1.0536	-
λ constant and μ Temp.	Exponential	3	-265.248	536.619	0.575	0.999	2.2038	-	1.2030	0.10578
λ Temp. and μ Temp.	Exponential	4	-264.955	538.114	2.070	0.9128	2.2776	-0.05523	1.0695	0.07642
λ Temp. and no μ	Linear	2	-272.381	548.824	12.779	0.999	1.6588	-0.23915	-	-
λ Temp. and μ constant	Linear	3	-265.748	537.618	1.573	0.999	2.1378	-0.26233	0.6189	-
**λ constant and μ Temp.**	**Linear**	**3**	**-264.961**	**536.044**	**0**	**Best**	**2.1238**	**-**	**0.9730**	**0.20179**
λ Temp. and μ Temp.	Linear	4	-264.575	537.355	1.310	0.3796	1.7996	0.28453	0.7834	0.47501

Mean parameter estimates presented for each model. Best-fitting model, as determined via a combination of the Akaike Information Criterion and likelihood ratio tests (see main text) highlighted in red and boldface. In our best-fit paleoaltitude-dependent model, speciation is positively exponentially correlated to Andean orogeny over time; adding extinction as a parameter did not improve model fit. Likewise, extinction is positively linearly correlated to temperature variation over time; allowing speciation to vary with temperature did not improve model fit. λ = speciation rate (in events/Ma/lineage); μ = extinction rate (in events/Ma/lineage); α = rate of variation of the speciation according to the relevant paleoenvironmental variable; β = rate of variation of the extinction according to the paleoenvironmental variable; NP: number of parameters in each model.

Table S5. Model comparison for the four BiSSE analyses presented in the main text.

**Andes (0: extra-Andean; 1: Andean)**											
**Model**	**NP**	**LogL**	**AICc**	**ΔAIC**	**P (LRT)**	λ_0	λ_1	μ_0	μ_1	q_{01}	q_{10}
Null model ($\lambda_0=\lambda_1$; $\mu_0=\mu_1$;$q_{01}=q_{10}$)	3	-361.737	729.597	15.332	0.99	2.932	2.932	2.592	2.592	0.084	0.084
$\lambda_0\neq\lambda_1$; $\mu_0=\mu_1$; $q_{01}=q_{10}$	4	-354.666	717.537	3.271	0.99	1.908	2.541	1.818	1.818	0.089	0.089
$\lambda_0=\lambda_1$; $\mu_0\neq\mu_1$; $q_{01}=q_{10}$	4	-357.126	722.457	8.192	0.99	2.713	2.713	2.621	2.164	0.102	0.102
$\lambda_0=\lambda_1$; $\mu_0=\mu_1$; $q_{01}\neq q_{10}$	4	-359.261	726.726	12.460	0.99	2.924	2.924	2.583	2.583	0.273	0.077
**$\lambda_0\neq\lambda_1$; $\mu_0\neq\mu_1$; $q_{01}=q_{10}$**	**5**	**-351.978**	**714.266**	**0**	**Best**	**1.267**	**2.975**	**1.152**	**2.275**	**2.275**	**2.275**
$\lambda_0\neq\lambda_1$; $\mu_0=\mu_1$; $q_{01}\neq q_{10}$	5	-352.912	716.133	1.867	0.99	1.690	2.477	1.636	1.636	0.043	0.109
$\lambda_0=\lambda_1$; $\mu_0\neq\mu_1$; $q_{01}\neq q_{10}$	5	-354.944	720.197	5.931	0.99	2.348	2.348	2.305	1.510	0.031	0.123
$\lambda_0\neq\lambda_1$; $\mu_0\neq\mu_1$; $q_{01}\neq q_{10}$	6	-351.892	716.219	1.953	0.678	1.292	2.938	1.184	2.224	0.081	0.088
**Elevation (0: low; 1: high)**											
**Model**	**NP**	**LogL**	**AICc**	**ΔAIC**	**P (LRT)**	λ_0	λ_1	μ_0	μ_1	q_{01}	q_{10}
Null model ($\lambda_0=\lambda_1$; $\mu_0=\mu_1$; $q_{01}=q_{10}$)	3	-370.173	746.468	16.468	0.99	2.994	2.994	2.656	2.656	0.175	0.175
$\lambda_0\neq\lambda_1$; $\mu_0=\mu_1$; $q_{01}=q_{10}$	4	-365.927	740.060	10.060	0.99	2.554	2.973	2.465	2.465	0.209	0.209
$\lambda_0=\lambda_1$; $\mu_0\neq\mu_1$; $q_{01}=q_{10}$	4	-367.173	742.551	12.551	0.99	2.944	2.944	2.822	2.480	0.206	0.206
$\lambda_0=\lambda_1$; $\mu_0=\mu_1$; $q_{01}\neq q_{10}$	4	-367.360	742.924	12.924	0.99	2.989	2.989	2.650	2.650	0.353	0.119
$\lambda_0\neq\lambda_1$; $\mu_0\neq\mu_1$; $q_{01}=q_{10}$	5	-360.316	730.942	0.942	0.99	0.963	3.566	0.740	2.876	2.876	2.876
**$\lambda_0\neq\lambda_1$; $\mu_0=\mu_1$; $q_{01}\neq q_{10}$**	**5**	**-359.845**	**730.000**	**0**	**Best**	**1.418**	**2.427**	**1.349**	**1.349**	**0.034**	**0.274**
$\lambda_0=\lambda_1$; $\mu_0\neq\mu_1$; $q_{01}\neq q_{10}$	5	-362.785	735.880	5.880	0.99	2.244	2.244	2.193	1.177	0.024	0.300

$\lambda_0 \neq \lambda_1$; $\mu_0 \neq \mu_1$; $q_{01} \neq q_{10}$	6	-358.759	729.952	*-0.048*	0.141	1.079	2.956	0.965	2.037	0.064	0.227
				** the lowest AIC is the model with 6 parameters, but it has a small ΔAIC as compared to simplest models*							
**Fruit type (0: capsule; 1: berry)**											
**Model**	**N P**	**LogL**	**AICc**	**ΔAIC**	**P (LRT)**	λ_0	λ_1	μ_0	μ_1	q_{01}	q_{10}
Null model ($\lambda_0=\lambda_1$; $\mu_0=\mu_1$; $q_{01}=q_{10}$)	3	-319.636	645.395	22.804	0.99	2.815	2.815	2.469	2.469	0.037	0.037
$\lambda_0 \neq \lambda_1$; $\mu_0=\mu_1$; $q_{01}=q_{10}$	4	-310.059	628.324	5.733	0.99	1.954	2.666	1.692	1.692	0.024	0.024
$\lambda_0=\lambda_1$; $\mu_0 \neq \mu_1$; $q_{01}=q_{10}$	4	-310.192	628.588	5.998	0.99	2.280	2.280	2.044	1.186	0.018	0.018
$\lambda_0=\lambda_1$; $\mu_0=\mu_1$; $q_{01} \neq q_{10}$	4	-319.270	646.745	24.155	0.99	2.814	2.814	2.467	2.467	0.030	0.052
$\lambda_0 \neq \lambda_1$; $\mu_0 \neq \mu_1$; $q_{01}=q_{10}$	5	-309.859	630.028	7.437	0.99	2.055	2.498	1.804	1.460	1.460	1.460
$\lambda_0 \neq \lambda_1$; $\mu_0=\mu_1$; $q_{01} \neq q_{10}$	5	-307.565	625.438	2.848	0.99	1.937	2.715	1.701	1.701	0.014	0.067
**$\lambda_0=\lambda_1$; $\mu_0 \neq \mu_1$; $q_{01} \neq q_{10}$**	**5**	**-306.141**	**622.591**	**0**	**Best**	**2.220**	**2.220**	**2.018**	**1.017**	**0.009**	**0.081**
$\lambda_0 \neq \lambda_1$; $\mu_0 \neq \mu_1$; $q_{01} \neq q_{10}$	6	-305.982	624.400	1.809	0.573	2.364	2.111	2.170	0.875	0.008	0.085
**Pollinator class (0: invertebrate; 1: vertebrate)**											
**Model**	**N P**	**LogL**	**AICc**	**ΔAIC**	**P (LRT)**	λ_0	λ_1	μ_0	μ_1	q_{01}	q_{10}
Null model ($\lambda_0=\lambda_1$; $\mu_0=\mu_1$;$q_{01}=q_{10}$)	3	-290.715	587.553	37.753	0.99	2.847	2.847	2.501	2.501	0.013	0.013
**$\lambda_0 \neq \lambda_1$; $\mu_0=\mu_1$;$q_{01}=q_{10}$**	**4**	**-270.798**	**549.800**	**0**	**Best**	**0.998**	**1.943**	**0.970**	**0.970**	**0.011**	**0.011**
$\lambda_0=\lambda_1$; $\mu_0 \neq \mu_1$; $q_{01}=q_{10}$	4	-272.780	553.766	3.966	0.99	2.051	2.051	2.045	1.144	0.007	0.007
$\lambda_0=\lambda_1$; $\mu_0=\mu_1$; $q_{01} \neq q_{10}$	4	-290.087	588.379	38.579	0.99	2.844	2.844	2.498	2.498	0.001	0.015
$\lambda_0 \neq \lambda_1$; $\mu_0 \neq \mu_1$; $q_{01}=q_{10}$	5	-269.357	549.023	*-0.777*	0.089	0.351	2.093	0.255	1.155	1.155	1.155

$\lambda_0 \neq \lambda_1$; $\mu_0 = \mu_1$; $q_{01} \neq q_{10}$	5	-270.113	550.534	0.734	0.242	0.990	1.952	0.968	0.968	0.007	0.021
$\lambda_0 = \lambda_1$; $\mu_0 \neq \mu_1$; $q_{01} \neq q_{10}$	5	-271.390	553.089	3.289	0.99	1.992	1.992	1.998	1.037	0.004	0.022
$\lambda_0 \neq \lambda_1$; $\mu_0 \neq \mu_1$; $q_{01} \neq q_{10}$	6	-269.330	551.094	1.294	0.23	0.377	2.083	0.288	1.140	0.016	0.019
				** the lowest AIC is the model with 5 parameters, but it has a small ΔAIC as compared to simplest models*							

Best-fitting model, as determined via a combination of the Aikake Information Criterion and likelihood ratio tests (LRT), is in red and bold text. The best model is first identified with the lowest AICc and then compared to other models with ΔAIC and LRT. The best-fitting models show that there is higher net diversification in the Andes (vs. extra-Andes), at high elevation (vs. low elevation), in berry-producing lineages (vs. capsular lineages), and in vertebrate-pollinated lineages (vs. invertebrate-pollinated lineages). Our second and third best models in each scenario also support these conclusions. λ = speciation rate (in events/Ma/lineage); μ = extinction rate (in events/Ma/lineage); q = transition rate between states; NP, number of parameter in each model.

Table S6. Correlated evolution of binary traits using BayesTraits.

**Maximum Likelihood**					
**Pair of Characters**	**LH (Dependent)**	**LH (Independent)**	**χ^2 statistic**	**df**	**p-value**
Pollination: Fruit	-56.583684	-60.354574	7.54178	4	0.109881
Pollination: Elevation	-104.072122	-107.581998	7.019752	4	0.1348481
Pollination: Andean	-88.546479	-92.452286	7.811614	4	0.09872793
Fruit: Elevation	-130.009089	-133.796692	7.575206	4	0.1084384
Fruit: Andean	-118.2341643	-122.1769823	7.8856359	4	0.09585848
Elevation: Andean	-159.696796	-168.894404	18.395216	4	0.001032828*

**Bayesian Inference**			
**Pair of Characters**	**Harmonic Mean (Dependent)**	**Harmonic Mean (Independent)**	**Bayes Factor**
Pollination: Fruit	-66.279838	-64.657858	3.24396
Pollination: Elevation	-111.382757	-111.797966	-0.830418
Pollination: Andean	-98.023314	-97.954698	0.137232
Fruit: Elevation	-138.288508	-138.146294	0.284428
Fruit: Andean	-127.542706	-126.737234	1.610944
Elevation: Andean	-229.079744	-172.277752	113.603984*

Results of likelihood ratio test (above) and Bayes factor of harmonic means from MCMC (below) for the correlated evolution of 6 pairs of binary characters. Tests were performed by comparing fit of a dependent v. independent model of trait evolution in BayesTraits. Asterisks mark traits whose evolution was found to be significantly correlated. LH: log-likelihood; df: degrees of freedom.

Table S7. Model comparison for the two diversity-dependence analyses presented, with mean parameter estimates for each model.

**Diversity Dependence**							
**Model**	**Mode of dependency**	**NP**	**logL**	**AICc**	**λ**	**µ**	**K**
λ depends on diversity (no extinction)	Exponential variation	2	-1056.369	2108.677	0.05975	-	31190.11
λ depends on diversity and µ constant	Exponential variation	3	-958.0664	1910.01	0.07683	3.041e-05	5873.402

Both models show that the group has not reached its ecological carrying capacity, suggesting that there currently is no limit to species diversity. λ = speciation rate (in events/Ma/lineage); µ = extinction rate (in events/Ma/lineage); K = carrying capacity; NP, number of parameter in each model.
